# Structural Investigation of a Novel N-Acetyl Glucosamine Binding Chi-Lectin Which Reveals Evolutionary Relationship with Class III Chitinases

**DOI:** 10.1371/journal.pone.0063779

**Published:** 2013-05-23

**Authors:** Dipak N. Patil, Manali Datta, Aditya Dev, Sonali Dhindwal, Nirpendra Singh, Pushpanjali Dasauni, Suman Kundu, Ashwani K. Sharma, Shailly Tomar, Pravindra Kumar

**Affiliations:** 1 Department of Biotechnology, Indian Institute of Technology Roorkee, Roorkee, India; 2 Department of Biochemistry, University of Delhi South Campus, New Delhi, India; 3 Central Instrumentation Facility, University of Delhi South Campus, New Delhi, India; Universidad de Granada, Spain

## Abstract

The glycosyl hydrolase 18 (GH18) family consists of active chitinases as well as chitinase like lectins/proteins (CLPs). The CLPs share significant sequence and structural similarities with active chitinases, however, do not display chitinase activity. Some of these proteins are reported to have specific functions and carbohydrate binding property. In the present study, we report a novel chitinase like lectin (TCLL) from *Tamarindus indica*. The crystal structures of native TCLL and its complex with N-acetyl glucosamine were determined. Similar to the other CLPs of the GH18 members, TCLL lacks chitinase activity due to mutations of key active site residues. Comparison of TCLL with chitinases and other chitin binding CLPs shows that TCLL has substitution of some chitin binding site residues and more open binding cleft due to major differences in the loop region. Interestingly, the biochemical studies suggest that TCLL is an N-acetyl glucosamine specific chi-lectin, which is further confirmed by the complex structure of TCLL with N-acetyl glucosamine complex. TCLL has two distinct N-acetyl glucosamine binding sites S1 and S2 that contain similar polar residues, although interaction pattern with N-acetyl glucosamine varies extensively among them. Moreover, TCLL structure depicts that how plants utilize existing structural scaffolds ingenuously to attain new functions. To date, this is the first structural investigation of a chi-lectin from plants that explore novel carbohydrate binding sites other than chitin binding groove observed in GH18 family members. Consequently, TCLL structure confers evidence for evolutionary link of lectins with chitinases.

## Introduction

The carbohydrate binding proteins from plants are usually called lectins, agglutinins, or hemagglutinins. Plant lectins are heterogeneous and highly diverse class of non-immune origin (glyco) proteins because of their carbohydrate-binding specificity, differences in molecular structure and biochemical properties [1]. For specific recognition, lectins have at least one non-catalytic domain, which reversibly bind to sugars or glycans of glycoproteins and glycolipids and do not modify the structure of carbohydrates [1], [2]. Lectins were first discovered in plants and later identified in organisms from all kingdoms of life [3]. Plant lectins are classified into twelve diverse families of evolutionary and structurally related lectin domains [2], [4]. Till now, it is an open question whether these twelve families form a closed or expandable group. Lectins show extensive structural diversity with the mutual orientations of the subunits in the tertiary folds giving rise to a variety of quaternary structures. These quaternary structures produce higher order sugar specificities, although oligomerization is not necessary for ligand recognition since single subunit of plant lectins were found to be able to bind carbohydrate [5]. Considering the structural architecture, plant lectins are grouped into seven folds [6]. There is no precise description for the biological function of the plant lectins because of their diverse classes and carbohydrate specificities. The lectins with homologous sequences also have different biological roles and their function cannot be generalized. Rigorous investigations need to be carried out for an understanding of the biological roles of individual lectins. Some functional characteristics of particular lectins reported in literature include antifungal [7], insecticidal [8], antiviral [9], antiproliferative, apoptosis-inducing [2] and symbiosis mediating between nitrogen fixing microorganisms and legume plants [3].

Another group that also encompasses the lectins is the glycosyl hydrolase family 18 (GH18) of chitinases. This family contains chitinase like lectins (chi-lectins)/proteins (CLPs) along with active chitinases. The CLPs are members of chitinase family in fold ((βα)_8_ barrel) while they might possess or lack chitinase activity. For example, *Parkia platycephala* lectin 2 (PPL2), an N-acetyl glucosamine (GlcNAc) binding lectin displaying chitinase activity is homologous to class III chitinases of the GH18 family [10]. The mammalian chi-lectins are also included in the family of GH18, which do not have chitinase activity. Some examples are YKL-40 (chitinase 3-like-1/HC-gp39/Chi3l1) [11] YKL-39 (chitinase 3-like-2) [12], mouse Ym1/2 (chitinase 3-like-3/4), stabilin-1-interacting chitinase like protein (SI-CLP) [13], SPX-40 proteins (SPG-40, SPC-40, SPS-40 and MGP-40) [14], [15], [16], [17] breast regression protein-39 (BRP-39, GenBank accession no. ABY53363) [18], rat cartilage glycoprotein (RATgp39, GenBank accession no. AF062038), oviduct-specific glycoprotein (NCBI Reference Sequence: NP_001073685.1). Some of these proteins are reported to have carbohydrate binding ability such as, 39-kDa human cartilage glycoprotein (HCgp-39), YKL-39, SI-CLP, SPS-40 and SPG-40 binds chitin fragment and Ym1 binds glucosamine and heparin/heparan sulfate. However, their physiological functions are not clearly understood [11], [19]. Some of these CLPs have specific function like XIP-I, a xylanase inhibitor protein I from *Triticum aestivum* [20] and XAIP, xylanase and alpha-amylase inhibitor protein from *Scadoxus multiflorus* [21].To gain further insight into structure-function relationship of such chitinase like lectins in general, GlcNAc binding glycoprotein, a tamarind chitinase like lectin (TCLL) was identified and analyzed by X-ray diffraction and biochemical & biophysical approach. Here, we present a high resolution crystal structure of TCLL and its complex with GlcNAc. TCLL displays significant sequence and structural similarity with members of GH18 family but the active site residues for chitinase activity along with chitin binding cleft residues were found to be mutated. So, TCLL lacks chitinase activity as well as binding to chitin polymer. The biochemical studies shows that TCLL is a chi-lectin and has specific carbohydrate recognition and different binding site than chitin binding site observed in the GH18 family chitinases. However, TCLL shows scant sequence and structural similarity with plant lectins and has different interacting residues with GlcNAc. Moreover, the structure depicts evolutionary relationship with class III chitinases and bestows the ability of plant to expand the sugar binding domain.

## Materials and Methods

### Ethics Statement

The human blood samples were collected and the experiments were performed with the understanding and written consent of the Institutional Human Ethical Committee (IHEC), Indian Institute of Technology Roorkee, Roorkee 247667, India. These blood samples were collected from the healthy volunteers with their written consent in the presence of Chief Medical Officer, Institute Hospital, IIT Roorkee and IHEC approved this consent procedure.

### Protein Purification and Determination of Oligomerization State

TCLL from tamarind seeds was purified by the protocol described previously [22]. To check the oligomerization state, TCLL was loaded onto a precalibrated Superdex-75 GL 10/300 (GE Healthcare) size-exclusion column with gel filtration calibration LMW standards on an AKTA Purifier system (GE Healthcare). The column was equilibrated with 100 mM Tris buffer, pH 7.4 and 1 ml fractions were collected. Peak fractions were resolved on 15% SDS-PAGE under non-reducing condition.

### Chitinase Assay

The protein purified in 0.1 M Tris-HCl pH 7.4 was extensively dialyzed and subsequently buffer exchanged into 50 mM sodium acetate pH 5. The chitinase assay was performed according to the protocol reported by Imoto and Yagishita [23]. The calcofluor white dye based assay for chitinase was also performed [24].

### Hemagglutination and Hemagglutination Inhibition Assay

Fresh 1 ml of human blood samples of different blood group (A, B, O) were collected in centrifuge tubes containing 6% EDTA as an anticoagulant in 3 ml of phosphate buffer saline (PBS), pH 7.2 and erythrocytes were obtained. For hemagglutination assay, 3% (v/v) suspension of human red blood cells in phosphate-buffered saline (pH 7.2) was prepared. Serial dilution of purified TCLL (50 µl) was taken in microtiter U-plate and was mixed with 50 µl of diluted erythrocytes cells. The plate was incubated at room temperature for 1 h. The specific activity was expressed as the minimum TCLL concentration (µg/ml) showing detectable hemagglutination activity. For the hemagglutination inhibition assay, N-acetyl glucosamine, N- acetyl galactosamine, cellobiose, carboxymethylcellulose, dextrose, glucose and galactose were used. The final concentrations of the additives were maintained at 10 mM before addition of red blood cells. The assay was monitored under microscope.

### Fluorescence Quenching Assays

Binding of the monosaccharides to TCLL was analyzed by ligand induced quenching of intrinsic tryptophan fluorescence according to the protocol described earlier [25]. Fluorescence measurements were carried out in a Varian Cary Eclipse fluorescence spectrometer. Emission spectra were recorded from 291–500 nm upon excitation at 290 nm. Excitation and emission slits were maintained at 5 nm and the scan speed was set at 100 nm/min. Standard reaction mixtures were prepared using 0.5 µM solution of protein in 25 mM phosphate buffer saline, pH 7.2 to a final volume of 1 ml. To study the binding affinity of monosaccharides (N-acetyl glucosamine, N-acetyl galactosamine, dextrose, glucose and galactose) to the enzyme, the additives were pre-incubated for 20 min and then spectra were obtained. The spectrum was corrected for background emission generated by buffer and ligands and the experiments were repeated in duplicates.

### Measurement of Binding Property by ITC

To measure accurately the binding affinity of TCLL to different sugars, isothermal titration calorimetry was employed to compare the binding affinity of GlcNAc, chitobiose, chitotetrose and chitohexose to TCLL. This was done by monitoring the energetics of molecular interactions at a constant temperature on iTC_200_ Isothermal titration calorimetry (GE Healthcare). The solutions were degassed to avoid air bubbles in the calorimeter during the experiment. The sample cell was filled with 0.3 mM protein solution and the injection syringe was filled with 8 mM sugar in 20 mM Tris (pH 7.4). The reaction was monitored for binding study with injections of 2 µl each for 10–20 injections having 100 sec of interval between them at 25°C. All the data were corrected for the heat of dilution produced by continued injection of ligand into the reaction cell. The data was fitted using ORIGIN software.

### Carbohydrate Estimation Assay and Characterization of Deglycosylated TCLL

The carbohydrate content of TCLL was quantitatively determined according to the phenol-sulphuric acid method [26] by plotting the standard curve using dextrose (0–25 µg). Experiment was done in triplicates and the percentage of carbohydrate content was estimated. The presence of carbohydrate was further confirmed by deglycosylating the protein chemically and investigating its properties. Pure TCLL (40 µg) was taken in glass vial and left overnight to evaporate. Subsequently, 5 µl of m-cresol was added to the dried protein on ice followed by addition of 20 µl of trifluoromethanesulfonic acid. Vials were capped and mixed properly performing the reaction in a fume hood. The reaction mix was incubated for 1 hr at 0°C. Reaction was stopped with 250 µl of cold acetone:N-ethylmorpholine (4∶1 by volume). Vial was then capped and mixed and solution was transferred to the microfuge tubes. Another 250 µl of acetone was added and mixed and divided equally in two microfuge tubes. Reaction was incubated for 30 min at −20°C and the protein was centrifuged for 10 min at 12000 rpm. Pellet was washed with 700 µl of acetone and centrifuged again. Pellet was subsequently dried and dissolved in 20 µl of 500 mM triethylammonium bicarbonate. Approximately 2 µg (2 µl) of the deglycosylated protein was electrophoresed on 15% SDS polyacrylamide gel [27].

### Intact Molecular Weight Determination by MALDI TOF

In order to get precise mass information for the intact protein, native and deglycosylated TCLL was reconstituted in 500 mM of triethyl ammonium bicarbonate at a concentration of 200 pmol/µl. Protein samples were further diluted to 3 fold in 50% acetonitrile and 0.1% tri-fluoro acetic acid. α-Cyano-4- hydroxycinnamic acid (CHCA) was used as a matrix for spotting the protein on to MALDI target plate mixed in 1∶1 ratio. Protein mass was measured in positive-ion linear mode over the mass range 10000–50000 Da. External mass calibration was performed using peaks of a mixture of cytochrome c (equine) at m/z 12,361.96, apomyoglobin (equine) at m/z 16,952.27, aldolase (rabbit muscle) at m/z 39,212.28, and albumin (bovine serum) at m/z 66,430.09 [28].

### Crystallization of TCLL and its Complex with GlcNAc

The high resolution crystals were obtained by various permutations and combination of crystallization conditions. Crystallization was performed by the vapor-diffusion method in 96-well sitting drop plates (Hampton Research, USA) at 293 K. The initial crystallization conditions were obtained with the sitting drop vapor**-**diffusion method using Crystal Screen (Hampton Research, USA). Crystals with maximum dimension were obtained within three days at 20°C using a precipitant solution containing 20 mM calcium chloride, 100 mM sodium acetate (pH 5) and 10–30% 2-methyl pentan 2,4-diol (MPD) with drop size of 1∶1 and 1∶2 with the reservoir buffer. For the complex formation, a soaking solution was prepared containing 20 mM calcium chloride, 100 mM sodium acetate (pH 5), 30% MPD and 20 mM N-acetyl glucosamine (GlcNAc). The complex was obtained by incubating TCLL crystals in the soaking solution for about 20 min at room temperature. Different sugars (N-acetyl galactosamine, dextrose, glucose and galactose) and chitin fragments (chitotriose, chitotetraose and chitohexaose) were soaked in similar manner with TCLL crystals but all trials were unsuccessful.

### Data Processing and Refinement

The X-ray diffraction data were collected at 1.49 Å for free TCLL and 1.6 Å for TCLL complexed with GlcNAc at the home source. The data were indexed and scaled using HKL2000 [29] and are summarized in Table 1. The structure of TCLL was solved by molecular replacement method using hevamine as a search model (PDB code: 2HVM). The transformed co-ordinates were subjected to 50 cycles of rigid body refinement followed by restrained refinement with REFMAC 5.2 from the CCP4i 6.3.0 package [30], [31]. The final refinement was done using the anisotropic temperature factors. The manual model building was carried out with graphics programs COOT [32]. The *F*_o_–*F*_c_ difference Fourier map generated indicated the presence of a MPD, a sodium acetate ion and a glycosylated site with two GlcNAc moieties in the free TCLL crystal. The complexed TCLL structure indicated presence of two additional GlcNAc moieties. The three dimensional models for MPD, acetate and GlcNAc were generated from PRODRG [33]. The models were evaluated using the program Molprobity [34]. The figures were generated using the program PyMOL [35] and ESPRIPT program [36].

**Table 1:** Crystallographic data and refinement statistics for TCLL and its complex with GlcNAc.

	TCLL	TCLL complex with GlcNAc
Crystallographic data		
Space group	*P*4_3_2_1_2	*P*4_3_2_1_2
Wavelength	1.54179	1.54179
Resolution (Last shell)	50–1.49 (1.5–1.49)	50–1.6 (1.63–1.60)
Cell dimensions		
* a* (Å)	100.1	100.1
* b* (Å)	100.1	100.1
* c* (Å)	81.7	81.8
Unique reflections (Last shell)	67001(2360)	54128 (1530)
Completeness (%) (Last shell)	98.8 (70.8)	98.1 (66.3)
R_sym_(%)^a^ (Last Shell)	4.3 (39.9)	3.4 (38.4)
I/σ (Last shell)	25.1 (2.2)	49.0 (3.0)
Multiplicity (Last shell)	5.5 (3.7)	7.7 (5.3)
Refinement	17.17–1.49	23.93–1.6
No. of reflections (working/test) No. of residues No. of non-hydrogen atom	63045 (59690/3355) 266 2248	51066 (48329/2737) 266 2279
Water molecules	432	381
*R*_cryst_ (%)	17.59	16.71
*R*_free_ (%)	18.42	19.83
Average *B*-factors (Å^2^) Chain A	17.31	19.31
Water atoms	37.22	38.68
All atoms	20.70	22.49
Ligand atom (TCLL-GlcNAc complex)	–	20.91
Wilson plot-B	27.69	30.84
rmsd on bond lengths (Å)	0.0072	0.0075
rmsd on bond angles (Å)	1.23	1.31
Ramachandran plot (%)		
Preferred	99.0	99.0
Allowed	1	1
Outliers	0.0	0.0

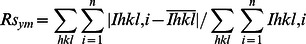

### Analysis of Crystallographic TCLL Sequence

The amino acid sequence of TCLL was proposed from the crystal structure solved at 1.49 Å. The program BLAST was used for detecting similar sequences available in database [37]. Multiple sequence alignment was performed using ClustalW [38] with the default parameters. Proposed sequence was analyzed for N-linked and O-linked glycosylation site using the Oxford Protein Analysis Linker (OPAL) available publicly through the Oxford Protein Production Facility UK (OPPF) website http://www.oppf.ox.ac.uk/opal/.

### Internal Sequence Determination Using Mass Spectrometry

For confirmation of amino acid sequence obtained by X-ray crystallography, LC MALDI TOF-TOF and ESI-Trap analysis of trypsin and Glu-C (V-8) endopeptidase digested TCLL protein (both native and deglycosylated) was performed. Peptides obtained by proteolytic digestion were separated on C-18 column and subjected to CID fragmentation for MS/MS sequence determination in both the instruments. The detailed methodology for internal sequencing is provided in Protocol S1 in Data S1.

### Phylogenetic Analysis

Phylogenetic tree for TCLL was constructed using MEGA version 5 program using the Neighbor-Joining method. The evolutionary distances were computed using the Poisson correction method [39].

## Results

### TCLL, a Monomeric, Glycosylated and GlcNAc Specific Chi-lectin

**Figure 1:**
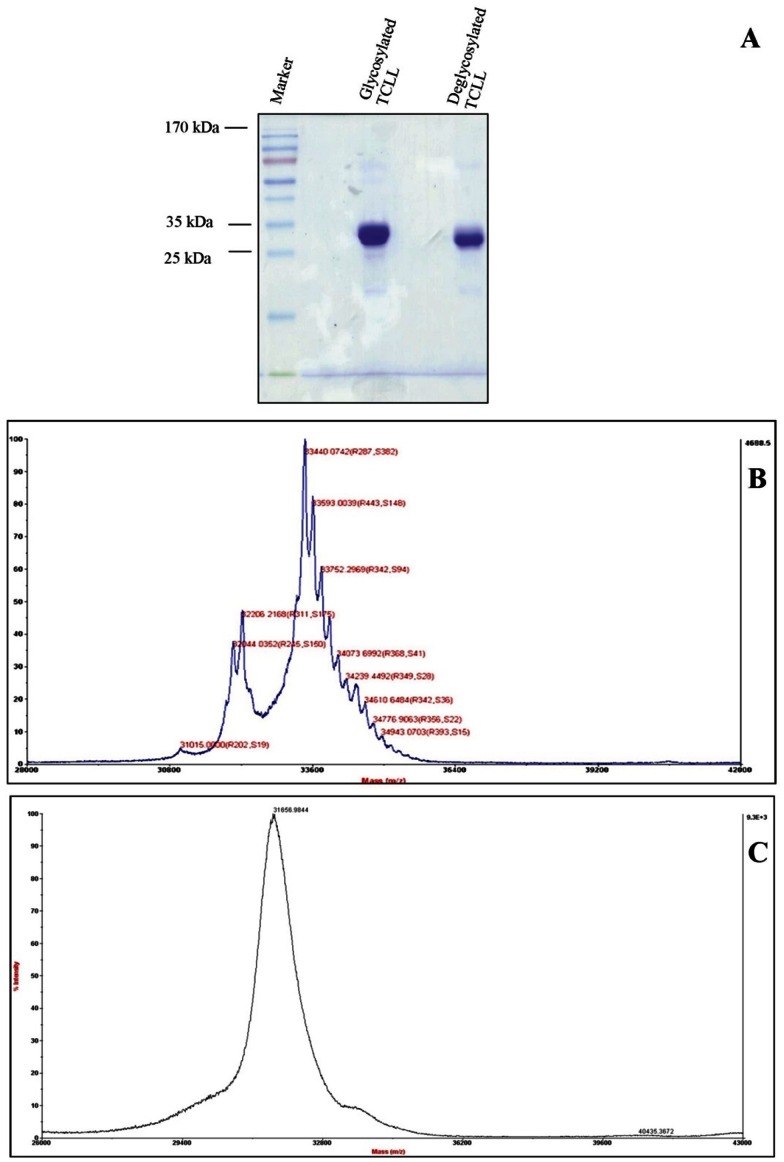
Characterization of native and deglycosylated TCLL. A. SDS-PAGE showing purity and difference in masses of glycosylated and deglycosylated TCLL. B. Intact mass spectrum of glycosylated TCLL with major species of molecular mass of 33,440 Da. Several other associated peaks indicate species with different carbohydrate content. An isoform of TCLL was also observed at 32, 206 Da. C. Intact mass spectrum of deglycosylated TCLL. It shows a single species indicating a single subunit and absence of sugar moiety. The molecular mass is also lower than its glycosylated native form as expected.

TCLL was purified from *Tamarindus indica* seeds by affinity chromatography and ion exchange chromatography. Gel filtration chromatographic analysis (data not shown) in combination with SDS-PAGE (Figure 1A) showed that it is a monomeric protein. This was further confirmed by intact molecular mass determination of native TCLL by MALDI TOF, which showed the major species at a molecular mass of 33440 Da along with the other minor glycoforms (Figure 1B). The phenol-sulphuric acid assay indicated that TCLL is a glycoprotein. The deglycosylated protein showed higher mobility on SDS-PAGE confirming the status of native TCLL as a glycoprotein (Figure 1A). MALDI TOF analysis of deglycosylated TCLL also showed a single major species of molecular mass 31811 Da, lower than that of native protein as expected (Figure 1C). All glycoforms observed in the native protein (Figure 1B) were absent in deglycosylated TCLL (Figure 1C). Comparison of molecular mass spectrum of glycosylated and deglycosylated TCLL (Figure 1B and 1C) thus indicated ten other glycoforms of the protein observed at m/z 33593, 33752, 33914, 34073, 34239, 34442, 34610, 34776, 34943 and 35113 Da.

**Figure 2:**
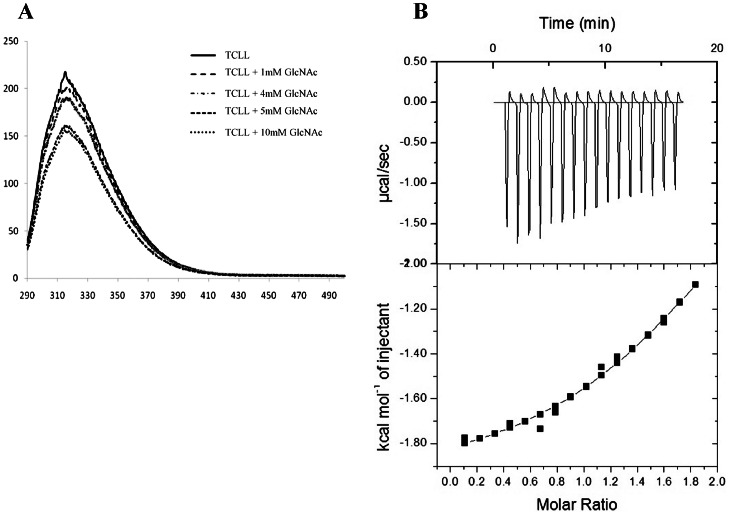
Effect of GlcNAc on the intrinsic fluorescence of TCLL and ITC analysis. A. Addition of GlcNAc to 0.5 µM solution of protein in 25 mM phosphate buffer saline, pH 7.2 at increasing concentration resulted in quenching of spectra. The emission spectra were recorded between 291–500 nm upon excitation at 290 nm. No shift in wavelength maxima from 316 nm was observed. B. Typical ITC thermograms and theoretical fits of the integrated peak to the experimental data for binding of TCLL with GlcNAc. The data (filled squares) were fitted nicely to a single binding sites model, and the solid lines represent the best fit.

Lectin-like activity was detected using human erythrocytes from three blood groups (A, B, O). TCLL showed the lattice formation of the erythrocytes of all the blood groups at a concentration of approximately 45 µg/mL as examined under the microscope. The formation of lattice was inhibited by 10 mM GlcNAc but not with other sugars tested. Further, binding studies of the sugar moieties was carried out by exploiting the intrinsic tryptophan fluorescence property of the protein. It was observed that addition of GlcNAc (1–20 mM) to a solution of protein resulted in quenching of the fluorescence between 310–320 nm without any shift of wavelength emission maxima (Figure 2). The fluorescence quenching occurred till 10 mM GlcNAc and beyond this concentration there was no detectable change in the spectra (Figure 2A). No other sugar showed any noteworthy change in the fluorescence intensity indicating that TCLL has affinity specifically for GlcNAc moiety. The binding activity of TCLL was analyzed for various polysaccharides of different lengths using ITC. It was found that only GlcNAc was fitted best in the curve that showed its binding to TCLL with Ka and Δ*H* values of 2.9×10^3^ M^−1^ and −2.6 kcal/mol respectively. The integrated data for GlcNAc binding were fitted nicely to a single binding sites model and the solid lines represent the best fit (Figure 2B). While the thermogram of chitobiose, chitotetrose and chitohexose to TCLL were not fitted to the experimental data that confirmed no interaction with them (shown in Figure S1) and its selectivity for GlcNAc.

### Quality of Model

Free TCLL structure was solved at 1.49 Å resolution with the primitive tetragonal space group *P*4_3_2_1_2 with cell dimensions of *a* = *b* = 100.1 Å, *c* = 81.7 Å by molecular replacement method using hevamine (2HVM) [40] as a search model. The crystals had one molecule in the asymmetric unit. TCLL structure refined with acceptable stereochemistry and final R_cryst_ and R_free_ values of 14.73 and 16.57%, respectively (Table 1). The final model showed coordinates for residues from 7 to 272. A total of 434 water molecules were identified in the model. Structure of TCLL in complex with GlcNAc was determined at 1.6 Å with space group same as the crystal structure of free TCLL. The model showed final R_cryst_ and R_free_ of 14.26 and 18.25%, respectively. Two sites were observed with GlcNAc moiety and it did not alter the overall conformation of the protein compared to the free structure. Crystallographic data and refinement statistics for structures of free and complex TCLL are summarized in Table 1.

### Sequence Analysis

**Figure 3:**
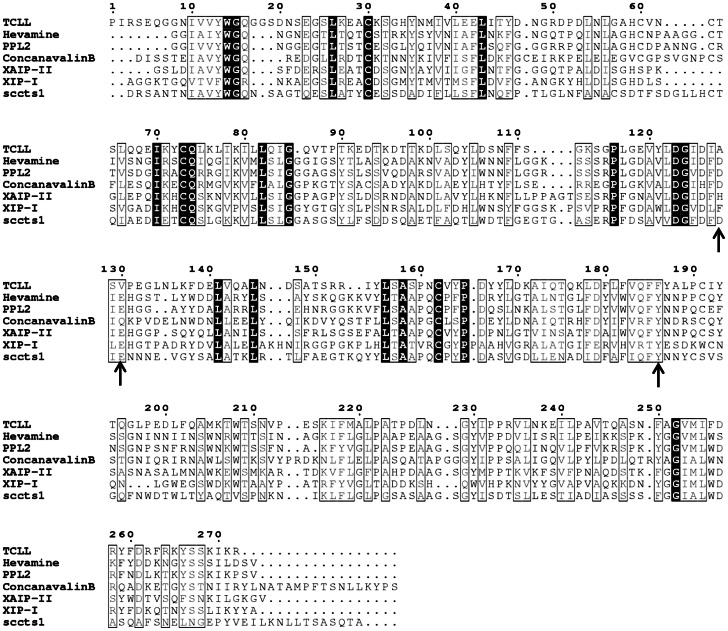
Sequence alignment of TCLL. Alignment of TCLL with GH18 family members such as hevamine, PPL2, concanavalin B, XIP-I, XAIP and SCCTS1. The alignment was done using the program CLUSTALW [38] and figure was prepared using ESPRIPT [36]. The conserved residues are represented in black background. The key active site residues for chitinase activity are shown by arrows.

TCLL sequence is proposed from crystal structure of TCLL solved at 1.49 Å. The sequence was further confirmed by mass spectrometry. Protease digested peptides were subjected to MALDI TOF-TOF and ESI Q-TRAP analysis and the crystallographic amino acid sequence was used as search template for the MS/MS sequence. Typical MS/MS spectra from TOF-TOF analysis and the consequent sequence of representative peptides are shown in Figure S2. Sequence coverage of ∼92% was obtained when results from both the mass spectrometric analysis were combined (ESI data not shown) and supported the crystallographic sequence (Figure 3).

TCLL showed significant sequence similarity to the reported class III chitinase of GH18 family in the pdb database searched using NCBI BLAST. The proposed amino acid sequence of TCLL, however, does not show any signature motif of family 18 chitinases which comprise of a sequence DXXDXDXE/[LIVMFY]-[DN]-G-[LIVMF]-[DN]-[LIVMF]-[DN]-x-E. This is due to natural variation in the signature motif in TCLL. A protein sequence BLAST against pdb database showed that TCLL shares sequence identity of 36%, 35%, 35%, 33% and 27% with hevamine (2HVM) from latex of *Hevea brasiliensis* [40], PPL2 (2GSJ) from *Parkia platycephala* seeds [10], concanavalin B (1CNV) from *Canavalia ensiformis* [41], xylanase and alpha-amylase inhibitor protein (XAIP) (3MU7) from *Scadoxus multiflorus* [21] and xylanase inhibitor protein I (XIP-I) (1OM0) from *Triticum aestivum* [42], respectively. It also shows 26% sequence identity with fungal “plant-type” family 18 chitinase, sccts1, from *Saccharomyces cerevisiae* (2UY2) [43] (Figure 3). Phylogenetic analysis also shows that TCLL is closely related to class III chitinases from plants of GH18 family and distinctly related to other chi-lectins from animals of GH18 (Figure S3). Proposed TCLL sequence analyzed for N-linked glycosylation, predicted 2 glycosylation sequon starting at positions 62 (NCT) and 146 (NDS).

### Overall Structure of TCLL

**Figure 4:**
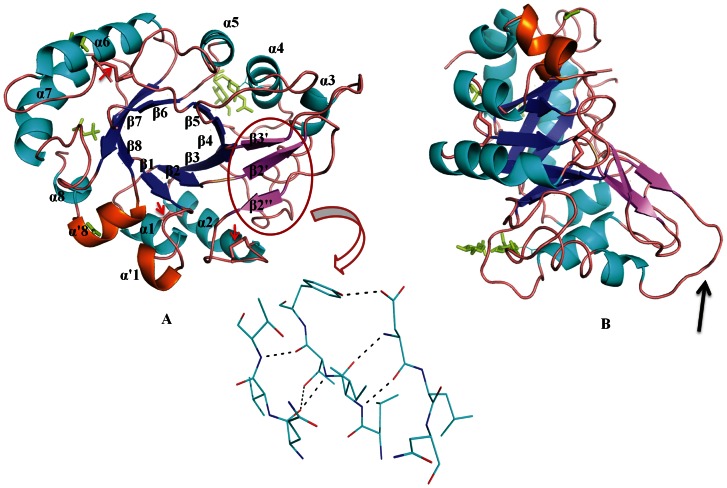
The crystal structure of TCLL. A Cartoon diagram representation of TCLL in (A) top view orientation and (B) side view orientation. The α-helices (α1 to α8) are shown in cyan and extra helices (α1 and α8) in orange. The β-strands (β1 to β8) are shown in TV blue and extra strands (β2′, β2′′ and β3′) in violet. Connecting loops are in wheat color. Two units of GlcNAc which is N-glycosylated at Asn146, two molecules of MPD and sodium acetate are shown in green color. Three disulphide bonds are indicated by red arrows. Unusual loop that protrudes out from domain are shown by black arrow in (B). The hydrogen bond network formed by extra strands is highlighted.

The overall structure of free TCLL, which comprises of a single polypeptide chain, consists of 266 amino acids, one molecules of MPD, two molecules of GlcNAc and sodium acetate. The TCLL model has an acceptable overall geometry (Table 1) and no residues in disallowed region of the Ramachandran plot. The structure displays a (βα)_8_ barrel topology as observed in the other GH18 chitinase family members [10], [40], [41]. Figure 4 illustrates nomenclature of α-helices and β-strands including the additional strands. Loops connecting carboxy terminus of strand to amino terminus of helix (8–12 amino acids long) and carboxy terminus of the helix to amino terminus of strand (4–8 amino acids long) were termed as βxαx and αxβx+1 loops, respectively, to maintain consistency in the nomenclature. The (βα)_8_ barrel is made up of eight core parallel β sheets (residues 10–13 (β1), 37–40 (β2), 80–84 (β3), 124–127 (β4), 155–158 (β5), 180–184 (β6), 216–222 (β7), 253–256 (β8)) surrounded by eight α-helices (26–32 (α1), 64–76 (α2), 97–101 (α3), 138–146 (α4), 170–175 (α5), 198–211 (α6), 233–246 (α7), 266–269 (α8)). Additionally, some distinct structural features in TCLL are: (1) one 23 amino acids long loop, β2α2, which contain an antiparallel β-hairpin (β2′ (43–46) & β2′′ (53–55)) stabilized by three hydrogen bonds. This extended loop is unusual for the (βα)_8_ topology. (2) Another loop β3α3 also contains insertion of β-sheet termed as β3′ (86–88) which makes parallel β-sheet conformation with β2′ of loop β2α2 (Figure 4) and this conformation is stabilized by two hydrogen bonds. Typical βαβ folding pattern is disrupted by additional short α-helix α1′ (20–23) & α8′ (258–264) (Figure 4). The insertion of additional α-helix was observed in several (βα)_8_ barrel topologies [41], [44].

Three disulphide bonds (Cys30–Cys73, Cys60–Cys63 and Cys162–Cys191) were observed, which are also present in hevamine, PPL2 and concanavalin B structures. These disulfide bridges help in maintaining the integrity of the enzyme. The helices α1 and α2 are connected by Cys30–Cys73 bridge, loops β5α5 and β6α6 by Cys162–Cys191 whereas Cys60–Cys63 bridge forms hairpin like structure in the loop β2α2. TCLL structure contains three well defined cis-peptides of which one is cis-proline (Tyr164–Pro165) and the other two cis-peptides are Glu41–Glu42 and Phe256-Asp257. Glycosylation was also observed clearly in electron density with two moieties of N-acetyl glucosamine binding to Asn146, one of the predicted glycosylation sites (Figure 4).

### N-acetyl Glucosamine Binding Site

**Figure 5:**
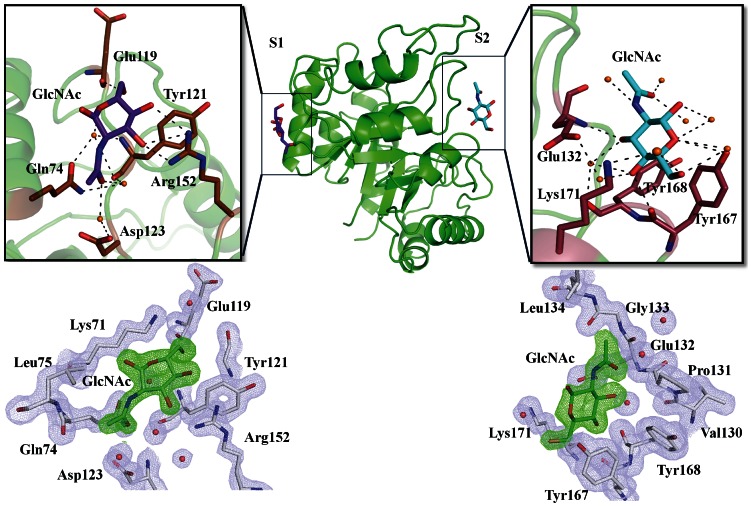
The crystal structure of the TCLL-GlcNAc complex and interactions of TCLL residues with GlcNAc. The TCLL-GlcNAc complex shows two GlcNAc sites denoted as S1 and S2. The S1 is formed by two loops, α3β4 and α4β5 and one helix α2 and S2 is formed by two loops β4α4 and β5α5 and one helix α5. GlcNAc binding sites are focused and interactions are shown. At S1, GlcNAc is stabilized through hydrogen bonds with residues Gln74, Tyr121, Asp123, Arg152 and three water molecules. At S2, GlcNAc is stabilized through hydrogen bonds with residues Glu132, Tyr167, Tyr168, Lys171 and eight water molecules in the pocket. Hydrogen bonds are shown in black dotted lines, water molecules in orange spheres, GlcNAc is shown as stick with purple blue carbons at S1 and cyan carbons at S2. Residues interacting with GlcNAc are shown as stick with brown carbons at S1 and wheat color carbons at S2. The 2Fo−Fc electron density maps are shown around 5 Å area of GlcNAc surroundings at S1 and S2 sites in TKI-PPT complex structure contoured at 1.0 σ.

The crystals of TCLL were soaked for upto 20 min in mother liquor containing 20 mM of different sugars. Interestingly, crystals soaked only with N-acetyl glucosamine displayed clear interpretable electron density at two different sites for GlcNAc. The 2Fo-Fc electron density map for GlcNAc and the interacting residues of TCLL at sites S1 and S2 is shown in Figure 5. Each site contain single molecule of GlcNAc and these sites are denoted as S1 and S2. These sites have similar type of polar residues, although the interaction pattern of GlcNAc atoms with these sites residues varies. The detailed interactions of pocket residues with GlcNAc are shown in Figure 5 and Table S1 in Data S1. Structural comparison between the free and the GlcNAc bound complex indicates that TCLL does not display any major conformational change upon binding of GlcNAc. The superposition of the ligand bound structure and the free structure showed an r.m.s. deviation of 0.06 Å (240 to 240 atoms).

In the TCLL-GlcNAc complex structure, S1 is formed by two loops, α3β4 and α4β5 and one helix α2. The GlcNAc molecule accommodates well in the pocket and is delimited by polar amino acids. GlcNAc atoms interact with two pocket residues (Glu119 and Arg152) directly and three pocket residues (Gln74, Tyr121 and Asp123) through three water molecules (Table S1 in Data S1). The site S2 is located in a shallow groove surrounded by two loops, β4α4 and β5α5 and one helix α5. Eight water molecules in the cavity form hydrogen bonds with GlcNAc moiety, among which three water molecules take part in stabilization by interacting with protein residues. The GlcNAc molecule interacts with Glu132 and Tyr167 directly and with Tyr168 and Lys171 through water. Comparison of the GlcNAc binding site S1 and S2 of TCLL shows that these pockets are surrounded by similar polar amino acids such as glutamate, tyrosine and arginine/lysine but the interactions of GlcNAc atoms with these amino acids differ drastically as summarized in Table S1 in Data S1.

### Substrate Binding Channel and Active Site Architecture for Chitinase Activity

The attempts to obtain crystals of the protein in complex with chitotriose, chitotetraose and chitohexaose were unsuccessful; the substrate binding site was examined and compared with the other GH18 family members. These subsite grooves are formed in TCLL by joining the carboxyl terminal residues of the β-strands with their subsequent loops. Compared to hevamine, the architecture of the subsites is different with some residues that interact with oligosaccharide being substituted. The subsite −4 in TCLL formed by loops β1α1 and β2α2 contains Gly18, Asn55 and Ala58 similar to hevamine residues Gly11, Asn45 and Gly48 indicating that this subsite in TCLL might accommodate the substrate. However, the existence of one extra helix α1′ (Asp20-Glu23) immediately above Gly18, might hamper the entry of chitooligosaccharide. The side chains of Ser19 and Asp20 protrude in the pocket with Gly18 hidden beneath Ser19 so that it might fail to attain the N-acetyl glucosamine moiety. The subsite −3 is present on the same loop as −4 is and includes Gln16 and Asn55 (Gln9 and Asn45 in hevamine). Two striking differences were observed in the TCLL −4 and −3 subsites as compared to hevamine: one is that these two subsites are very close to each other (the C^α^ distance between Gly18 and Gln16 is 5.5 Å compared to 6.6 Å of corresponding hevamine residues) and second that the cavity formed by loops is wider than hevamine; so most likely it may not able to grip two GlcNAc moieties. As for subsite –2, it is the most disturbed site. The loop forming this groove - β3α3 - is very different in TIM barrel topology with extra β-strand (β3′). It protrudes straight away from core barrel rather than forming pocket and seems to be flung out from (βα)_8_ fold. However, in hevamine, strand β3 moves little inside in the barrel and the corresponding loop is projected in the groove and arranged in such a way that it holds chitin molecule by interacting with it. The residues Val87 and Thr88 on one side of the groove on the extra β3 strand and Phe256 on the opposite side of the groove from this subsite in TCLL are substituted (natural variants) as compared to hevamine Gly81, Ile82 and Trp255 residues. The C^α^ distance between Val87 and Phe256 is 21.7 Å, which is very high as compared to that in hevamine (11.9 Å) and forms very shallow and broad cavity. The residue Thr88 participates in hydrogen bond interaction with Thr45 of strand β2′ which is parallel to β3′ and is involved in stabilization of strand rather than participation in accepting GlcNAc moiety. Thr88 is also engaged in hydrogen bonding with Asp47 so that overall this groove is completely unsuitable and may not hold chitin molecule.

**Figure 6:**
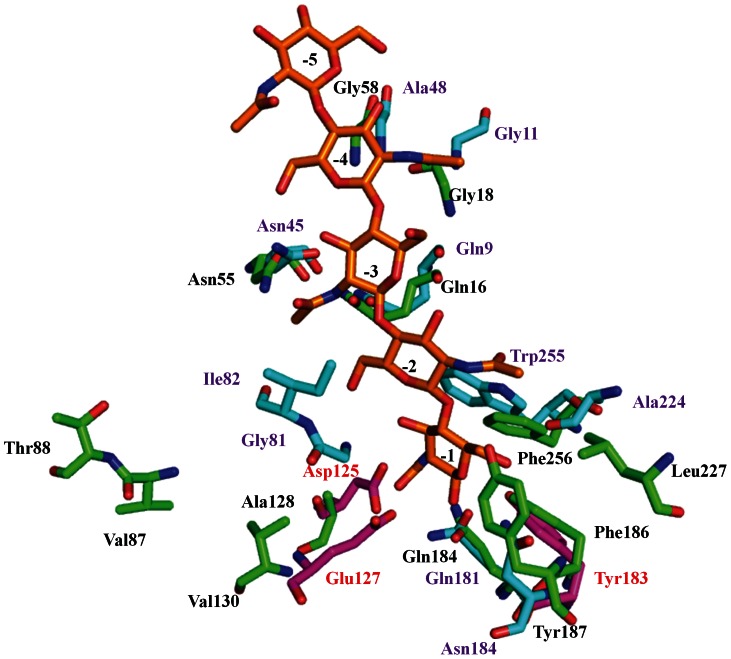
Substrate binding subsites and active site residues of TCLL. Hevamine (2HVM and 1KQY) was superimposed onto TCLL and the interacting residues with GlcNAc moiety are shown. Hevamine residues are in cyan sticks with purple label and the corresponding TCLL residues are in green sticks with black label. The active site residues of hevamine are shown in pink sticks with red label and corresponding residues of TCLL are Ala128, Val130 and Phe186 show mutations of key catalytic residues.

The most crucial subsite −1 contains Gln184, Tyr187, Leu227 and Val87 that correspond to Gln181, Asn184, Ala224 and Gly81 of hevamine. Tyr187 protrudes in the cavity and reduces the ability to bind GlcNAc. The architecture of the loop that contains Ala224 in hevamine is such that it bends toward the core barrel and is involved in making cavity while Ala224 form hydrogen bond with O6 of GlcNAc moiety. This type of interaction is not possible in the TCLL structure because the loop moves away from barrel and the residue Leu227 is not able to interact with O6 of GlcNAc and also has rigid β-turn conformation. The C^α^ distance between Val87 and Leu227 is 25.5 Å, whereas the corresponding distance in hevamine is 12.3 Å. Overall, this subsite is also not favorable for binding to chitin molecule. The key residues for catalytic activity are Asp125, Glu127 and Tyr183 in hevamine. Glu127 is considered to donate a proton to glycosidic bond [45], [46], [47]. Asp125 stabililizes the transition state during hydrolysis and Tyr183 assist Asp125 in making hydrogen bond to carbonyl oxygen of the N-acetyl group. It has been shown that mutation of the very important catalytic residue glutamate (Glu127) in hevamine impaired the chitinase activity [45], [48]. The respective residues in TCLL are Ala128, Val130 and Phe186. Neither Ala128 nor Phe186 side chain is able to make hydrogen bond with GlcNAc residue to stabilize the oxazolium intermediate. The most important residue glutamate is substituted to valine that cannot donate a proton to the scissile bond. The loop that bears Ala128 and Val130 has different orientation than the corresponding loop in hevamine. Corresponding residues of the active site and substrate binding site of TCLL and hevamine are shown in Figure 6. The differences explained here in TCLL structure rules out any possibility of chitin binding as well as chitinase activity.

### Structural Comparison with Other GH18 ‘Plant Type’ Chitinases

The three dimensional structure of TCLL superimposed onto those of hevamine, PPL2, concanavalin B, XAIP, XIP-I and sccts1 which shares 36%, 35%, 35%, 33%, 27% and 26% sequence identity delivers a root mean square deviation (r.m.s.d.) of 0.79 (for 195 C^α^ atoms), 0.76 (for 194 C^α^ atoms), 0.8 (for 190 C^α^ atoms), 0.83 (for 185 C^α^ atoms), 0.97 Å (for 192 C^α^ atoms) and 0.82 (for 149 C^α^ atoms), respectively. This suggests that TCLL displays an overall fold similar to those of the above structures except some loop deviations (Figure S4). Compared to hevamine, some structural features in TCLL are different - such as glycosylation at Asn146, substitutions in the substrate binding site and the active site, smaller sizes of strand β1 and helix α3 than corresponding hevamine strand and helix, different conformation and extra strands of loops β2α2 and β3α3, loop β3α3 obtrusion from (βα)_8_ fold forming hinge like conformation and some major divergence of loops β4α4, β7α7 and α3β4. Asp123 & Trp179 are strictly conserved in all members of family 18 plant chitinases [40]. While aspartate is conserved, tryptophan is replaced by phenylalanine in TCLL. Unlike PPL2, which is an endochitinase with N-acetyl glucosamine binding hemagglutinin, TCLL shows glycosylation and lacks endochitinase activity due to substitution of active side residues. Nevertheless TCLL is N-acetyl glucosamine binding lectin that shows lattice formation of human erythrocytes. Comparison of GlcNAc binding sites of TCLL with PPL2 demonstrates that PPL2 has similar type of residues at corresponding site of S1 pocket and can bind GlcNAc. The variation was observed at loop β4α4 of S2 pocket, which is longer and forms cavity. The residues of S2 that hold the GlcNAc are also substituted in PPL2. The corresponding cavity in PPL2 is shallow and might not interact with GlcNAc.

Concanavalin B, XIP-I and XAIP belongs to GH18 family as they preserved some characteristic features of this family [21], [41], [42] but are devoid of chitinase activity due to substitution of key residues of active site. Narbonin from the seeds of *Vicia narbonensis* and *Vicia pannonica* is also reported as a member of this family that lacks chitinase activity [49], [50], [51]. Narbonin has Glu132 at similar position to hevamine catalytic glutamate and still do not show chitinase activity as Glu132 is engaged with Arg87 in forming salt bridge. One extra disulphide bond in concanavalin B Cys41–Cys93, which is engaged in stabilization of the loops β2α2 and β3α3 is observed that is unusual for the GH18 family [41] whereas, XIP-I and XAIP shows only two disulphide bonds [21], [42]. When compared to XIP-I which is a dual inhibitor of xylanase belonging to GH10 and GH11 family [20], loop α4β5 in TCLL is shorter in length. The elongated loop observed in XIP-I enter into the cavity of GH11 xylanase and residue Arg149 interact direct with active site residues Glu85 and Glu176 while such interacting residues are missing in TCLL. GH10 xylanase also has a TIM barrel topology and is inhibited by XIP-I helix α7 (232–245) that blocks central part of substrate binding groove (−3 to +2). The residues Lys234 interact directly with Glu131 and through water with Glu239. In TCLL, the corresponding helix residues are substituted. Other interacting residues 225 to 232 display different conformations and this loop moves towards core barrel rather than protrude out. Residues 189 to 205 attained some amino acid insertions and the resultant longer loop adopts β-turn conformation that obstructs the interaction with helix α7. Overall, TCLL might not function like XIP-I. XAIP shows GH11 xylanase and GH13 α-amylase inhibition. Kumar S. *et al,* (2010) [21] proposed loop α3β4 for xylanase inhibition and loop β6α6 and helix α7 for α-amylase inhibition. In case of TCLL, loop α3β4 is shorter in length and loop β6α6 has different conformation and proposed residues of helix α7 interacting with α-amylase are substituted. Alignment of TCLL structure with that of sccts1 shows difference at some loop regions and the active site region [43]. Although, several features typical of family 18 are preserved in TCLL structure such as the (βα)_8_ barrel fold with the two family18 consensus regions (but with some mutations at these regions), presence of two non-proline cis-peptide bonds (Table S2 in Data S1), three disulphide bonds and elongated loop β2α2 having one antiparallel β-hairpins is novel.

### Structural Comparison with Other GH18 Chitinase Like Proteins

A DALI search [52] shows structural similarity of TCLL with those of GH18 chitinase like proteins (CLPs) or chi-lectins except for some loops and α/β insertion domains. Several reports demonstrated that CLPs show high sequence similarity to GH18 family but lack chitinase activity. They are thought to perform variety of functions and interact with sugars [53], [54], [55], [56], [57]. There was lack of significant sequence similarity between any CLPs and TCLL and structure of TCLL superimposed on those of HCgp-39 [53], Ym1 [19], 40 kDa goat mammary gland protein (MGP-40) [17], and imaginal disc growth factor-2 from *Drosophila melanogaster* (IDGF-2) [58], YKL-39 [12], SI-CLP [13], SPC-40 [15], SPS-40 [16] and SPG-40 [14] showed overall similar structural fold but quite high r.m.s. deviation. However, CLPs share some common features with TCLL like conserved three cis-peptide bonds and mutations in signature motif DXXDXDXE. TCLL is a glycoprotein like most of CLPs, however; glycosylation is not common to GH18 and the glycosylation site in TCLL is different than that observed in CLPs like HCgp-39, MGP-40, SPX-40 proteins, YKL-39 and IDGF-2. Comparison of chitin binding cleft of TCLL with other CLPs shows TCLL has smaller and open cleft and a deep pocket like architecture (Figure S5).

TCLL structure compared to the structure of HCgp-39 in complex with chitin fragment (1NWT, 1HJW) [11], [53] shows differences in chitin binding groove. The residues that putatively interact with chitin are substituted in TCLL. The groove formed in HCgp-39 is long and deep allowing it to accommodate chitin fragment faultlessly. However, in case of TCLL, chitin binding cavity is shorter and very superficial. Moreover, the cavity forming loops on the opposite sides are very apart thus resulting in an open cavity (Figure S5). The two important residues, Trp99 and Trp352, of HCgp-39 which engage in hydrophobic stacking interactions as well as hydrogen bonds with sugars [11] and contribute in ligand recognition are missing in TCLL. The structure of chitin fragment bound HCgp-39 suggests that chitin, which is structural element of nematodal and fungal pathogen to human, can be physiological ligand of HCgp-39. Further, HCgp-39 is known to switch on innate immunity by sensing chitin of pathogen and cooperating with macrophage [53].

YKL-39 is a member of GH18 family without any chitinase activity due to lack of active site residues. It is reported that it binds chitooligosaccharides through its conserved tryptophan residues. It has been shown that the chitinase activity of YKL-39 can be retained by just substitution in acive site residues with catalytic residues required for chitinase activity [12]. Comparison of TCLL structure with YKL-39–(GlcNAc)_6_ complex structure shows that substitution of conserved residues reported to have interaction with chitin polymer at chitin binding groove. There are also some loop region differences are observed which hold the GlcNAc polymer. The corresponding residues Tyr104, Tyr146, Asp213, Tyr269 and Trp360 which makes interaction with GlcNAc in YKL-39 structure are replaced with Gln86, Pro131, Tyr187, Thr224 and Phe256 in TCLL. The respective cleft forming loops, which hold Tyr269 and Tyr104 in YKL-39, are far apart from each other in TCLL making an open cavity. Apart from those aromatic residues, Trp36 and Trp218 observed in sugar binding cleft are also substituted in TCLL. YKL-39 has retained the ability to bind chitin polymer and can be reverted back to active chitinase by substituting with catalytic residues at active site and hence called as pseudo-chitinase. In case of TCLL, major differences lie at chitin binding cleft could be the reason for loosing chitin binding property.

Another 40 kDa glycoprotein, SPG-40 is reported to have GlcNAc polymer binding property but no catalytic activity observed due to the mutation of a catalytic Glu residue to Leu. The SPG-40 structure with different GlcNAc polymer shows that it has a long well formed carbohydrate-binding groove composed of aromatic residues which shows stacking interaction with sugar ligands. [14] Structural comparison of TCLL with SPG-40 shows that the respective interacting residues with sugars and aromatic residues of the groove are substituted in TCLL. The major differences are also observed in loop regions which form the groove that could be the reason for making distorted groove in TCLL. Another member of GH-18 family, SI-CLP contains a typically wide, more open and saddle-shaped sugar binding cleft. The longer and negatively charge cleft may be the reason behind the broad ligand recognition of SI-CLP. The conserved aromatic amino acids of the cleft are also present in SI-CLP. ITC analysis revealed that SI-CLP has the highest binding activity for GlcNAC tetramer and LPS binding was shown by SPR in SI-CLP [13]. Structural comparison of TCLL with SI-CLP shows that large differences at the cleft forming loops make distorted cavity in TCLL. Conclusively, structural comparison of TCLL with CLPs, which have chitin binding ability shows that TCLL has substitution in the solvent-exposed aromatic residues. These hydrophobic residues form stacking interaction with sugars and they are conserved in HCgp-39, YKL-39, SI-CLP, SPS-40 and SPG-40 and their extensive interaction with chitin shows that these residues are important for its binding. Previous studies have also indicated that mutagenesis of the conserved tryptophan leads to the reduction or complete loss of activity [59], [60], [61]. In case of SI-CLP, it has been shown that the mutation of these aromatic residues of the cleft drastically reduces the binding activity to GlcNAC tetramer [13].

The structural studies of other CLPs (Ym1, IDGF-2 and SPC-4) also do not show any chitin binding ability. Ym1, a secretory novel protein from murine activated peritoneal macrophages also shares structural similarity with TCLL. Ym1 has glucosamine and heparin/heparan sulfate binding ability but other significant biological functions have not yet been revealed. The chitin binding groove of Ym1 is neither distinct (Figure S5) nor conserved,yet it binds glucosamine and the binding site is observed inside the core of TIM barrel at the carboxy terminal of the β-strands. In case of TCLL, this site is not properly defined, shallow and interacting residues were not found conserved. One another member of this family is IDGF-2 from *Drosophila melanogaster*. The structure of IDGF-2 shows partly blocked binding cavity that lacks a proper configuration of residues for binding to oligosaccharides (Figure S5). However, it has been proposed that it might promote cell proliferation by assisting insulin. The structure of SPC-40 also shows GH18 chitinase like fold but has no catalytic activity. This protein has some conserved aromatic amino acids at the chitin binding cleft which are reported for saccharide binding. However, they are unable to bind to chitin polymer due to some variations in the neighbouring residues and change in the shape of its cavity [15]. Structural superimposition of TCLL with SPC-40 proteins shows high r.m.s. deviation and large variation in loop regions. Comparison with chitin binding groove also shows major differences in the lining residues and loop variation, which makes unique shape cleft in TCLL (Figure S5).

These structural comparisons reflect that GH18 chitinase like proteins preserve the TIM barrel domain of the family with no chitinase activity and they have evolved to participate in diverse functions. TCLL has TIM barrel domain without a well-defined chitin binding groove and lacks the catalytic activity. However, it has binding affinity for GlcNAc that explains the evolutionary progression from chitinase to chitinase like lectin. The biological significance of GlcNAc binding of TCLL, however, needs to be explored further.

### Comparison with Other GlcNAc Binding Lectins

As TCLL is GlcNAc binding chi-lectin, we evaluated the structure of other GlcNAc binding proteins. For example, *Agaricus bisporus* lectin (ABL) (PDB: 1Y2X) [62], *Boletus edulis* lectin (BEL) (PDB: 3QDV) [63], *Sclerotium rolfsii* lectin (SRL) (PDB: 2OFE) [64], *Ulex europaeus* lectin II (PDB: 1QOO) [65], *Psathyrella velutina* lectin (PVL) (PDB: 2C4D) [66], wheat germ agglutinin (WGA) (PDB: 2UVO) [67], and two lectins which are specific for saccharides containing GlcNAc namely, *Urtica dioica* agglutinin (UDA-VI) (PDB: 1EHH) [68] and *Phytolacca americana* lectin (PL-D2) (1ULM) [69]. Structurally, TCLL does not display any similarity with the aforesaid GlcNAc binding proteins. Further, the GlcNAc interacting residues and environment of the cavity were compared. ABL has two diverse binding sites that differentiate in to different configurations at a single epimeric hydroxyl site. The GlcNAc interacted mainly with residues Asp79, Ile80, Thr82, Arg103 and Tyr113 of ABL. BEL has two sites: site 1 that hold the GalNAc and T-antigen disaccharide while site 2 is most likely chitin binding site having preference for GlcNAc and chitobiose. The interacting residues of BEL with GlcNAc are Asp78, Val79, Thr81, Arg102 and Tyr113. Asp78 defines specificity for binding to GlcNAc as its carboxylate oxygens form hydrogen bonds with O4 and O6 of the GlcNAc. SRL is similar to ABL and BEL with two distinct carbohydrate-binding sites, a primary and a secondary site. GalNAc is attached at the primary while GlcNAc prefers only the secondary site. Residues Asp77, Ile78, Thr80, Arg101, and Tyr112 are involved in hydrogen bonds with GlcNAc moiety. ABL, BEL and SRL have common interacting residues that delineate specificity to GlcNAc. The interactions of GlcNAc O3 and O4 with tyrosine, O4 and O6 with aspartate and O5 and O6 with arginine are the common specificity pattern in ABL, BEL and SRL. In TCLL-GlcNAc complex, such types of interactions are not observed (Table S1 in Data S1).

*Ulex europaeus* lectin II contains promiscuous carbohydrate-binding site with triad of Asp86/Gly106/Asn136 that accommodates GlcNAc, galactose and fucosylgalactose. PVL binds GlcNAc at six different sites with minor differences in binding mode. Two hydrogen bonds are formed by the side chain of one asparagine (or aspartate) residue with the O3 and O4 of GlcNAc. The O3 also develops a hydrogen bond to a conserved tryptophan. Hydrophobic contacts with GlcNAc are made by conserved histidine and tyrosine at each site. WGA has four hevein domains in each polypeptide. Structure of WGA complexed with GlcNAc displays five binding sites and residues (Asp or Glu/Ser/Ser/Tyr) of cavity built by hevein domains of 36-kDa homodimer interact with GlcNAc moiety. UDA-VI has two hevein analogous domains. The complex structure of UDA-VI with NAG3 displays two binding sites of UDA-VI molecule. One NAG3 is sandwiched between two molecules of UDA-VI. The hevein domain residues Trp21/Trp23and Ser19/Cys24/Tyr30 of domain 1 and His67/Trp69 and Ser65/Tyr76 of domain 2 recognize and interact with NAG3 molecule. PL-D2 also has two hevein domains (I and II) and the interacting residues in its complex with NAG3 are Ser20, Trp22, Tyr24 and Tyr31 of domain I and Trp43, Ser61, Tyr63, Trp65 and His72 of domain II. Taken together, comparison with other GlcNAc binding lectins shows that TCLL has different architecture of cavity and novel binding mode, which has not been documented so far in other plant lectins and chilectins.

## Discussion

Based on the significant sequence identity, TCLL seems to be a member of the class III chitinase of GH18 family. The crystal structure of TCLL also shows similar (βα)_8_ TIM barrel topology as those of GH18 family members. However, it has some distinct structural features which are unusual for the (βα)_8_ topology such as long β2α2 loop which contain an antiparallel β-hairpin. The other unusual loop β3α3 also revealed insertion of a β-sheet (Figure 4). A single glycosylation site with two units of GlcNAc was also observed in TCLL structure. Glycosylation is very rare in plant GH18 chitinases of class III. Only few chitinases from this family were reported to have glycosylation such as suspension-cultured bamboo [70], XIP-I [42], mammalian chitinase like proteins (MGP40, HCgp-39, YKL-39) [17], [53] and *Drosophila melanogaster* protein (IDGF-2) [58]. However, the glycosylation site observed in TCLL is distinct compared to the other related proteins.

The GH18 family members with chitinase activity, have a conserved catalytic triad of Asp, Glu and Tyr residue in the active site (Asp125, Glu127 and Tyr183 in hevamine), whereas, the corresponding residues observed in TCLL are Ala128, Val130 and Phe186. Therefore, the loss of chitinase activity in TCLL could be due to the mutation of polar residues to non-polar residues in the active site. Some other members of chitinase-like family of proteins also show the lack of chitinase activity with varying mutations of these three essential residues; although some of them show binding to saccharides/polysaccharides in a similar manner as observed in chitinases [11], [17], [19], [21], [41], [42], [51], [58]. In TCLL, the evaluation of the chitin binding cavity shows that it is shorter and has a deep pocket like architecture instead of groove and the cavity forming loops are distantly apart, which make it an open cavity. Comparison of TCLL with chitinases and CLPs shows that it has substitution of important residues responsible for chitin binding at chitin binding groove. The biochemical studies show no binding to chitin polymer. The co-crystallization and soaking experiments of TCLL with chitotriose, chitotetraose and chitohexaose were also unsuccessful, which indicated that TCLL might have inappropriate chitin binding cavity. The ITC experiments show that thermograms for the binding interaction of chitobiose, chitotetrose and chitohexose with TCLL were not fitted to the experimental data, which confirmed that TCLL has no binding affitiniy with these sugars. It is noteworthy to state that TCLL has unique cavity shape that may not accommodate the GlcNAc polymer.

Furthermore, the biochemical studies of TCLL revealed its hemagglutination property and binding to *N*-acetylglucosamine. It indicated that TCLL is a GlcNAc binding chi-lectin. The ligand bound structure of TCLL with GlcNAc further supported that TCLL is GlcNAc specific chi-lectin. The two GlcNAc binding sites (S1 and S2) observed in the TCLL complex structure are novel as compared to other lectins. The binding pattern of S1 and S2 residues with GlcNAc is also different. The interacting residues, architecture of cavity and novel binding mode makes TCLL distinct from other GlcNAc binding lectins. Collectively, it revealed that TCLL is a chitinase like GlcNAc specific lectin. There were also some other similar proteins (tamarinin and chitinase like proteins) reported to have chitinase activity from tamarind seeds [71], [72]. Interestingly, the sequence alignment of these chitinase like proteins from tamarind showed that TCLL has 41% and 39% sequence identity with tamarinin and chitinase, respectively. Moreover, tamarinin and chitinase showed only 63% sequence identity with each other. The intact mass spectrum of TCLL also shows presence of some minor isoforms (Figure 1B). These observations indicate that chitinase and chitinase like proteins are present in tamarind. The presence of several chitinases and chitinase like proteins/genes were also reported in the fruit fly, *Drosophila melanogaster* [73], malaria mosquito, *Anopheles gambiae* [74] and the red flour beetle, *Tribolium castaneum* [75]. Therefore, TCLL is an independent protein present with different features in tamarind seeds along with other chitinase/chitinase like proteins.

The evolution of TCLL might have occurred in tamarind sequentially. This evolutionary pathway most likely involved gene duplication events with mutations at active site leading to TCLL, a GlcNAc binding chi-lectin. The sequence analysis of TCLL, chitinase and tamarinin could be an evidence to the conversion of a plant chitinase into a chi-lectin (Figure S6). Similar evidence of analogous evolutionary mechanisms have also been reported earlier in plants [76], mammals [77] and insects [73]. The gene duplication events, followed by mutations at catalytic site of mammalian GH18 family chitinase proteins gave rise to the evolution of broad spectrum chi-lectins [77]. For example, mammalian chi-lectins HCgp-39 [53] and the murine chitinase like lectin, Ym1 [19] display (βα)_8_ barrel fold similar to GH18 chitinases but lacks chitinase activity. HCgp-39 binds chitin fragment and Ym1 shows specificity towards GlcN and heparin/heparan sulfate [19]. Hitherto the physiological significance of these findings are not clear. A lectin (RobpsCRA) reported from the bark of *Robinia pseudoacacia* shares 50% sequence identity with plant class V chitinases of the GH18 family. This protein also lacks chitinase activity but can interact with high-Man N-glycans. The experimental structure has not been solved, however, molecular modeling has shown the protein to have a TIM barrel domain [76]. Recognition of TCLL as a chi-lectin, which evolved from a chitinase, illustrates acquiring of new activity by losing chitinase activity. Adoption of a new function by inactive chitinases of class III of GH18 family was also reported earlier. For example, XIP-I which is an inactive chitinase was reported to inhibit fungal xylanases of class GH10 and GH11 [20]. XAIP is documented as inhibitor of xylanases of family GH11 and α-amylases of family GH13 [21]. These evolved proteins still had the same fold as that of parent proteins but developed a different site or conformation of interacting loops with ligands. Interestingly, these are not just inactive chitinases but proteins having precise biological functions. So utilization of family GH18 existing scaffold is common to conquer new function, as also exemplified by TCLL. The topology of TCLL also shows structural affirmation of the fact that legume plants are capable of building a domain or site for specific identification of carbohydrate using existing scaffolds. It is notable that no attempts were made to reclassify these chitinase like proteins of GH18 family which has defined functions. The categorization of the CPLs into a specific class having similar role could provide easier ways to study their evolutionary link in depth as their evolutionary relationship and physiological role are still unsolved.

To the best of our knowledge, this is the first structural report of plant CLPs which binds GlcNAc monomer at the surface of TCLL at two different sites instead of the chitin binding site of GH18 family. The relevant question arises here about the biological role of TCLL in the seeds and the significance of GlcNAc binding. It is documented that several plant lectins serve as plant defence proteins due to their carbohydrate specificity and capability to fight against phytophagous insects and fungi [78]. It was also observed that lectins from seeds of Leguminosae family play role in symbiosis with nitrogen fixing bacteria [3], [78]. Additionally, in case of TCLL it was also observed that it is degrades during germination as found in most of the seed lectins [78]. Considering the presence of specific carbohydrate binding site and other observations, it could be conferred that TCLL is not just a seed storage protein. Additional exploration is required to ascertain the physiological function of the GlcNAc binding TCLL. The elucidation of the specific recognition of GlcNAc also needs further investigation.

### Atomic Coordinates

The coordinates and structure factors of refined model for TCLL structures have been deposited in the Protein Data Bank with accession numbers for TCLL (4B15) and TCLL-GlcNAc (4B16).

## Supporting Information

Figure S1 **The ITC analyses showed no binding of chitin polymers to TCLL.** The thermogram of chitobiose (A), chitotetrose (B) and chitohexose (C) to TCLL were not fitted to the experimental data which shows no interaction of these polymers with TCLL.
(TIF)

Figure S2 **Typical MS/MS spectra from MALDI TOF-TOF analysis and corresponding sequence of representative peptides.** CID MS/MS spectra of trypsin and Glu-C endoproteinase digested TCLL obtained from MALDI TOF/TOF mass spectrometer. A. MS/MS spectrum of m/z 1420.61. B. MS/MS spectrum of m/z1862.92. C. MS/MS spectrum of m/z 911.43. D. MS/MS spectrum of m/z 1389.61.
(TIF)

Figure S3 **Phylogenetic analysis of TCLL.** The tree was constructed by Neighbor-Joining method using MEGA5 program and the evolutionary distances were computed using the Poisson correction method.
(TIF)

Figure S4 **Superposition of TCLL with homologous structures of the GH18 family.** The ribbon diagram shows the superposition of C^α^ atoms of TCLL (red) (4B15), hevamine (blue) (2HVM) from latex of *Hevea brasiliensis*, PPL2 (yellow) (2GSJ) from *Parkia platycephala* seeds, concanavalin B (green) (1CNV) from *Canavalia ensiformis*, xylanase inhibitor protein I (magenta) (XIP-I) from *Triticum aestivum*, xylanase and alpha-amylase inhibitor protein (cyan) (XAIP) from *Scadoxus multiflorus* (3MU7) and sccts1 from *Saccharomyces cerevisiae* (orange) (2UY2). The superposition shows that the overall structure is conserved except some loop regions shown by arrows.
(TIF)

Figure S5 **Electrostatic surface potential map of TCLL with other chi-lectins.** TCLL (4B15), HCgp-39 (1HJW), Ym1 (1E9L), YKL-39 (4AY1), SI-CLP (3BXW), SPG-40 (2DSZ), SPC-40 (2DPE), SPS-40 (2DSU) and IDGF-2 (1JND) displaying chitin binding groove. Electrostatic potential was calculated by Pymol and is colour-coded on the surface from blue (∼63) to red (∼63). Only HCgp-39, YKL-39, SI-CLP, SPS-40 and SPG-40 has appropriate groove and chitin fragment binds at this groove. TCLL displays more negative site and has deep pocket like structure. Ym1, SPC-40 and IDGF-2 do not have well defined cavity.
(TIF)

Figure S6 **Sequence alignment of TCLL with other chitinase/chitinase like protein from tamarind.** Alignment of TCLL with tamarinin and chitinase from tamarind. The conserved residues are represented in black background and the key active site residues for chitinase activity are represented by arrows. The alignment was done using the program CLUSTALW and figure was prepared using ESPRIPT.
(TIF)

Data S1 (DOC)
