# Supplementary figures and images for: Structural Investigation of a Novel N-Acetyl Glucosamine Binding Chi-Lectin Which Reveals Evolutionary Relationship with Class III Chitinases
Source: PLoS One. 2013 May 23;8(5):e63779. doi: 10.1371/journal.pone.0063779 (PMC3662789; doi:10.1371/journal.pone.0063779)

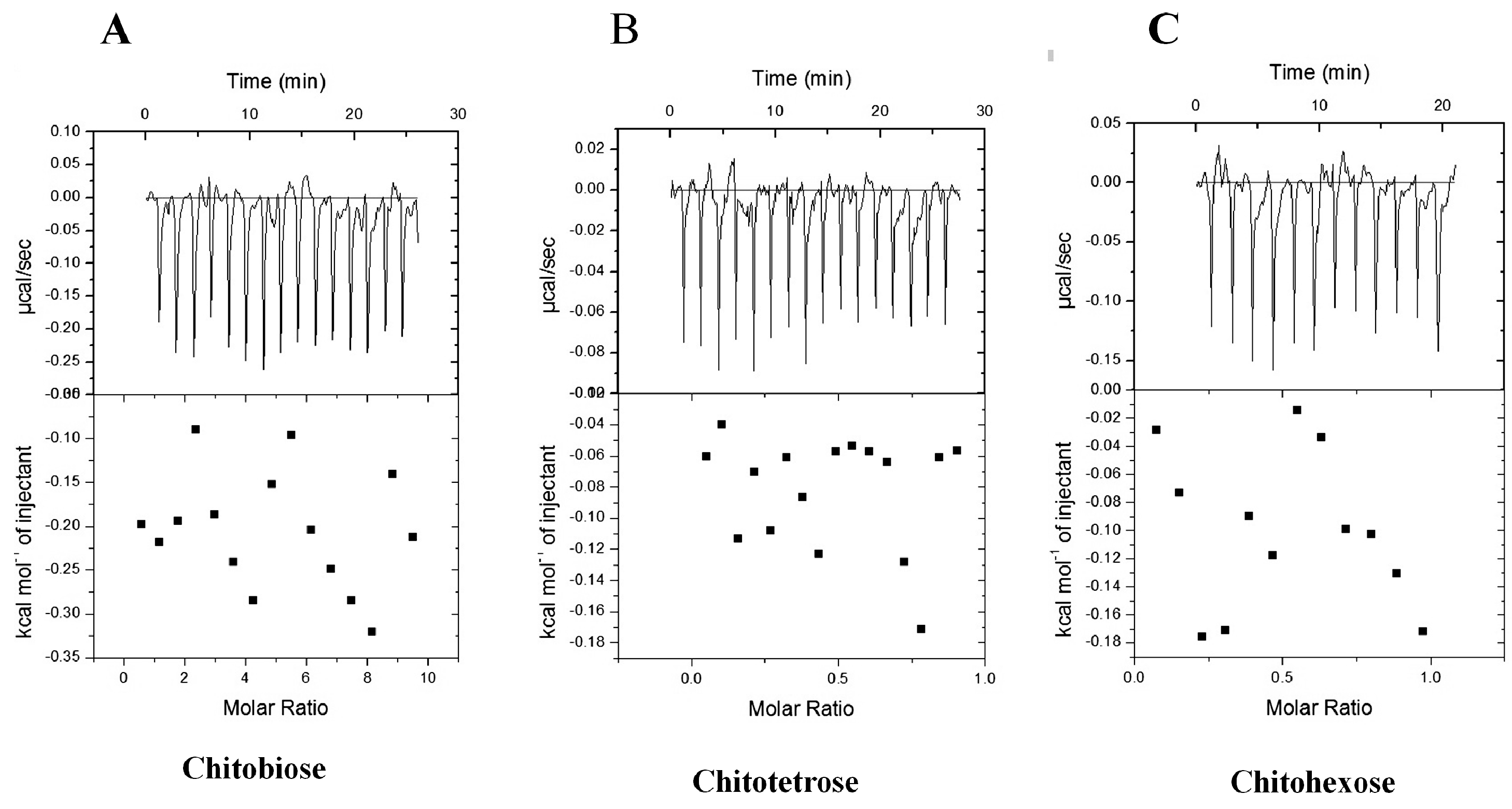

Supplement: Figure S1 — The ITC analyses showed no binding of chitin polymers to TCLL. The thermogram of chitobiose (A), chitotetrose (B) and chitohexose (C) to TCLL were not fitted to the experimental data which shows no interaction of these polymers with TCLL. (TIF) [file pone.0063779.s001.tif]

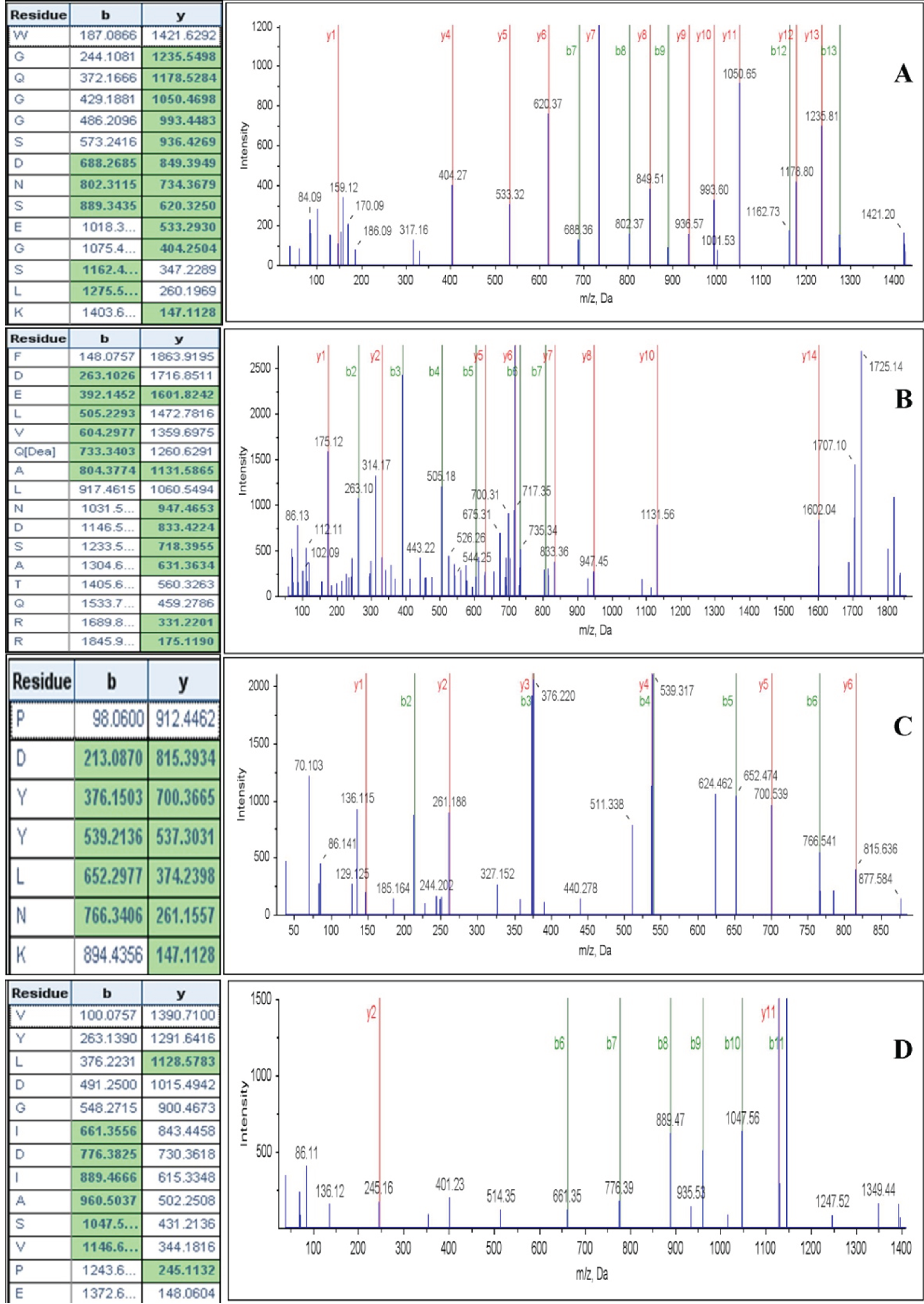

Supplement: Figure S2 — Typical MS/MS spectra from MALDI TOF-TOF analysis and corresponding sequence of representative peptides. CID MS/MS spectra of trypsin and Glu-C endoproteinase digested TCLL obtained from MALDI TOF/TOF mass spectrometer. A. MS/MS spectrum of m/z 1420.61. B. MS/MS spectrum of m/z1862.92. C. MS/MS spectrum of m/z 911.43. D. MS/MS spectrum of m/z 1389.61. (TIF) [file pone.0063779.s002.tif]

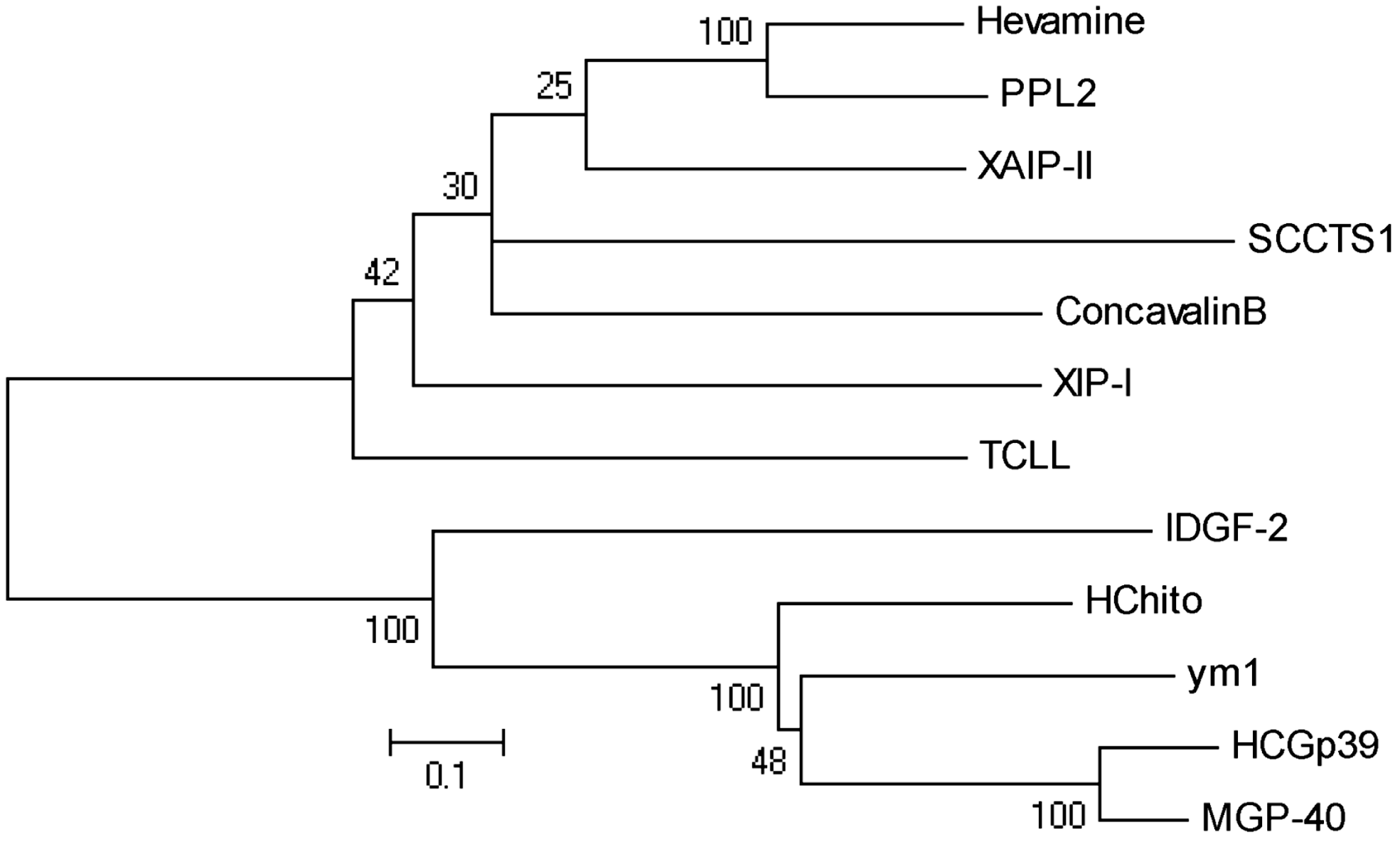

Supplement: Figure S3 — Phylogenetic analysis of TCLL. The tree was constructed by Neighbor-Joining method using MEGA5 program and the evolutionary distances were computed using the Poisson correction method. (TIF) [file pone.0063779.s003.tif]

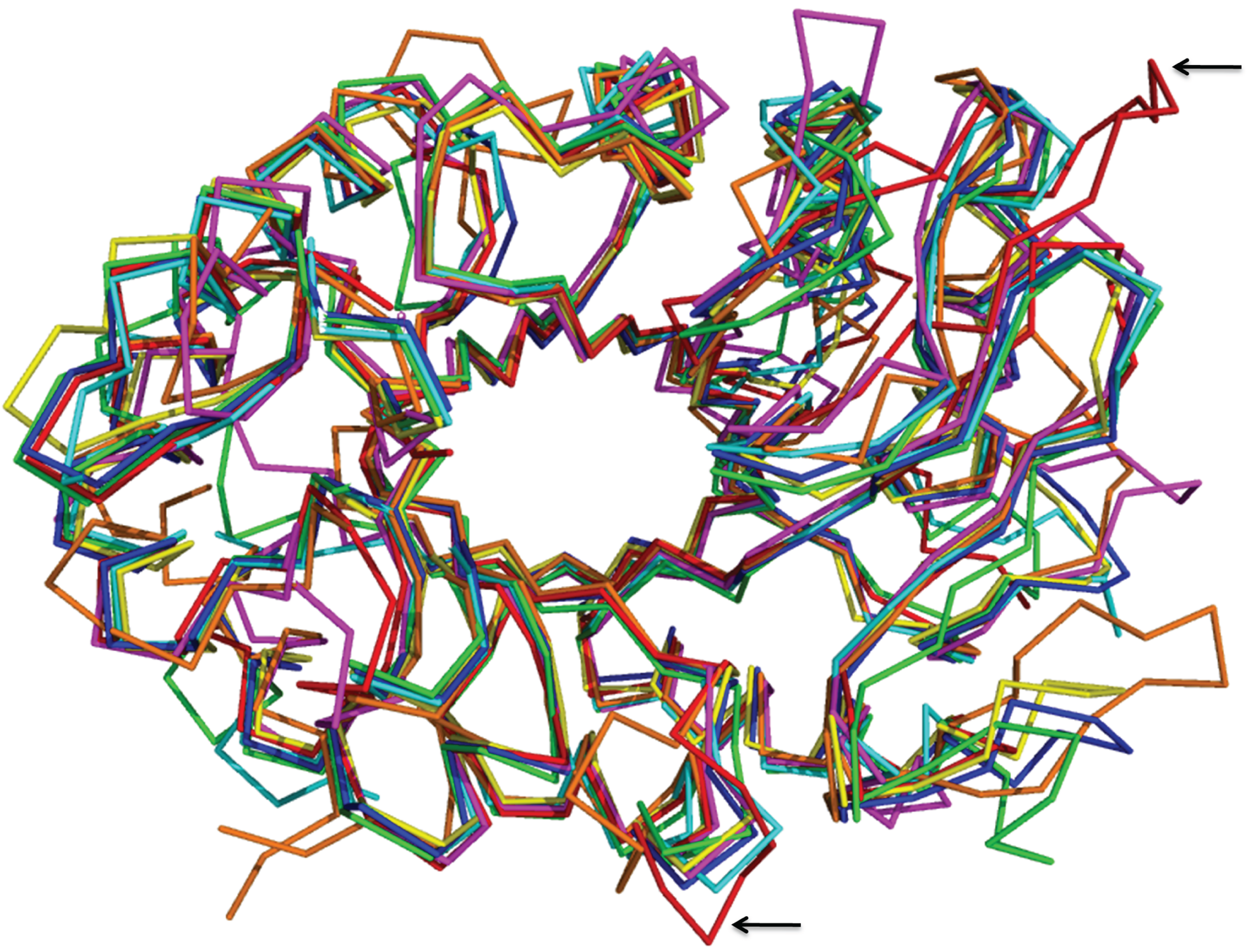

Supplement: Figure S4 — Superposition of TCLL with homologous structures of the GH18 family. The ribbon diagram shows the superposition of Cα atoms of TCLL (red) (4B15), hevamine (blue) (2HVM) from latex of Hevea brasiliensis, PPL2 (yellow) (2GSJ) from Parkia platycephala seeds, concanavalin B (green) (1CNV) from Canavalia ensiformis, xylanase inhibitor protein I (magenta) (XIP-I) from Triticum aestivum, xylanase and alpha-amylase inhibitor protein (cyan) (XAIP) from Scadoxus multiflorus (3MU7) and sccts1 from Saccharomyces cerevisiae (orange) (2UY2). The superposition shows that the overall structure is conserved except some loop regions shown by arrows. (TIF) [file pone.0063779.s004.tif]

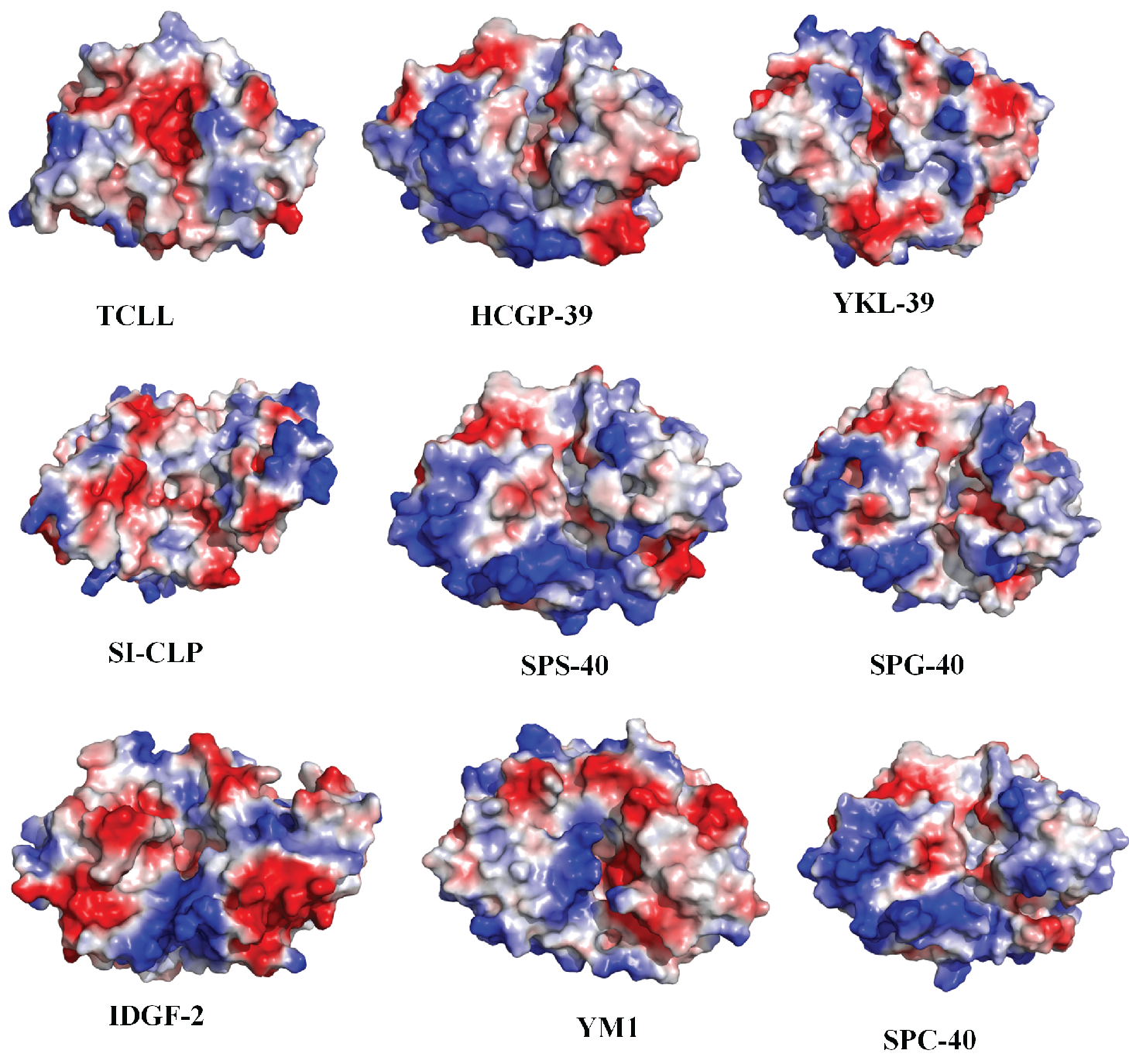

Supplement: Figure S5 — Electrostatic surface potential map of TCLL with other chi-lectins. TCLL (4B15), HCgp-39 (1HJW), Ym1 (1E9L), YKL-39 (4AY1), SI-CLP (3BXW), SPG-40 (2DSZ), SPC-40 (2DPE), SPS-40 (2DSU) and IDGF-2 (1JND) displaying chitin binding groove. Electrostatic potential was calculated by Pymol and is colour-coded on the surface from blue (∼63) to red (∼63). Only HCgp-39, YKL-39, SI-CLP, SPS-40 and SPG-40 has appropriate groove and chitin fragment binds at this groove. TCLL displays more negative site and has deep pocket like structure. Ym1, SPC-40 and IDGF-2 do not have well defined cavity. (TIF) [file pone.0063779.s005.tif]

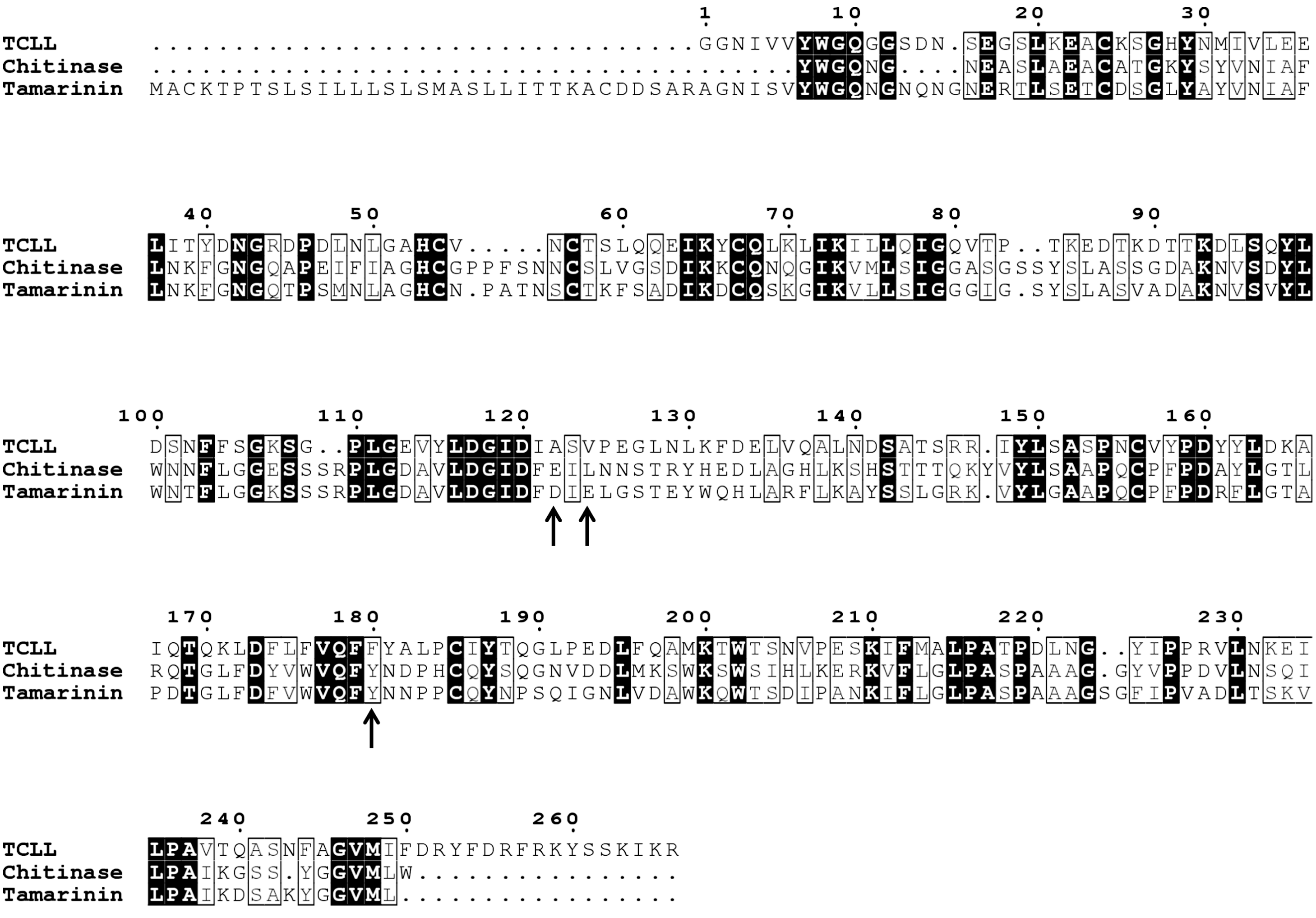

Supplement: Figure S6 — Sequence alignment of TCLL with other chitinase/chitinase like protein from tamarind. Alignment of TCLL with tamarinin and chitinase from tamarind. The conserved residues are represented in black background and the key active site residues for chitinase activity are represented by arrows. The alignment was done using the program CLUSTALW and figure was prepared using ESPRIPT. (TIF) [file pone.0063779.s006.tif]
