# Supplementary material for: Structural Investigation of a Novel N-Acetyl Glucosamine Binding Chi-Lectin Which Reveals Evolutionary Relationship with Class III Chitinases
Source: PLoS One. 2013 May 23;8(5):e63779. doi: 10.1371/journal.pone.0063779 (PMC3662789; doi:10.1371/journal.pone.0063779)
Supplement: Data S1 — (DOC) [file pone.0063779.s007.doc]

**Supplementary Data S1**

**Supplementary Protocol S1:**

Coomassie Blue-stained protein bands were excised from the corresponding SDS-polyacrylamide gels and destained with 50% acetonitrile in 50 mM ammonium bicarbonate (ABC) for 1 h with three intermittent changes of the solution. The supernatants were replaced with 10 mM DTT (Sigma) in 50 mM ABC solution to reduce the proteins for 15 min at 56 ºC. The supernatant was discarded and 20 mM iodoacetamide (Sigma) in 50 mM ABC was added for 15 min at at 25 ºC in the dark to alkylate the proteins. The gel pieces were collected, washed three times with 200 µl of 50 mM ABC for 15 min at RT and then dried *in vacuo*. The gel pieces were rehydrated with 3.0 µl of 12.5 ng/µl of sequencing grade modified trypsin gold (Promega) and 3 µl of 50 ng of V8 protease in 50 mM ABC in two separate reactions and incubated for 60 min at 25 ºC. Further 50 µl of 10% acetonitrile in 50 mM ammonium bicarbonate was added and the digestion of proteins was continued for 18 h at 37 ºC with agitation. The supernatant was collected and the gel pieces were extracted for 40 min at 37 ºC with successive 50 µl aliquots of 0.1% trifluoroacetic acid and 50% acetonitrile. The combined extracts were concentrated using Speed Vac.

The peptide extracts were reconstituted in 40 µl of 98% Water, 2% Acetonitrile and 0.1% TFA. The aliquot of 12 µl of digested peptide was separated on Chromolith CapRod Monolithic capillary column (150 mm × 0.1 mm RP-18 endcapped). The LC gradient for the separation of the peptides was prepared from two solvent systems: solvent A (98% Water, 2% Acetonitrile and 0.1% Trifloroacetic acid) and Solvent B (98% Acetonitrile, 2% Water and 0.1% Trifluoroacetic acid). Trypsin and V8 protease digested peptides were desalted with solvent A for 30 min on Cap-trap C18 column at a flow rate of 20 µl in the reverse direction. After desalting, the peptides were eluted from the column over 60 min using gradients of solvent A and B in following steps: 5-10% B in 7 min, 10-35% B in 18 min, 35-50% B in 10 min, 50-90% B in 2 min followed by a washing step for 5 min at same gradient. Most of the peptides eluted between 10 min to 30 min. All the eluted peptides were simultaneously mixed with α-cyano-4-hydroxycinnamic acid (CHCA) matrix at a ratio of 1:1 and spotted after every 7 sec on LC MALDI plate.

The LC MALDI plates spotted with peptides as above were analyzed on AB Sciex 4800 Plus TOF/TOF analyzer in reflector ion mode. To identify the peptide and its PTM modification peptide masses and their MS/MS sequences derived from the mass spectrometric analysis were searched for matches using the Protein Pilot 2.0 software against given X-ray crystal structure sequence. In the Protein Pilot search, biological modification and amino acid substitution was incorporated as ID focus factor along with the gel based ID search. Detected protein threshold was fixed at confidence score of 99.9%. For LC/MS/MS Q-trap analysis, samples were prepared as follows. Denaturant was added to the sample tubes containing 5µg of protein, followed by addition of reducing reagent and incubation at 60 ºC for 1 h. Cysteines were then blocked as above for 10 min followed by tryptic digestion overnight at 37 ºC. The digested peptides were extracted with acetonitrile: water (50:50) and vacuum dried in speed vac.

Peptides obtained as above were reconstituted in 0.1% formic acid in the ratio of 3: 97 acetonitrile: water solution before subjecting to 1D Nano LC (1200 Series, Agilent Technologies) for peptide separation on Enrichment column and reverse phase (RP) column. The LC was further coupled to Nano ESI-LC/MS system (4000 Q TRAP LC/MS/MS system, Applied Bisosytems MDS SCIEX) for peptide ionization and detection. The latter was equipped with a nanoelectrospray ionization source (Nanosource II, Applied Bisosytems MDS SCIEX) and fitted with a 15 µm fused silica emitter tip (New Objective, Woburn, MA). RP LC was performed on a 3.5 µm (75 µm × 150 mm) ZORBAX 300 SB-C18 Nano LC column (Agilent Technologies,Germany) with a 5 µm (300 µm x 5 mm) ZORBAX 300 SB-C18 Nano LC column (Agilent Technologies, Germany) in place. Samples were loaded onto a trap or guard column in a volume of 5–10 µl and were equilibrated for 5 min in 97% solvent A, 3% solvent B at a flow rate of 10 µl/min. Solvent A is 0.1% formic acid in water and solvent B is acetonitrile. On switching on line with the MS, a linear gradient at 300 nL/min from 97 to 45% solvent A was developed for 40 min and in the following 5 min the composition of the mobile phase was decreased to 10% A before increasing to 97% A for a 20 min equilibration before the next sample injection. MS data were acquired automatically using Analyst 1.4.2 software (Applied Biosystems MDS SCIEX, Concord, Canada). An EMS survey scan was conducted over 400–1600 amu, followed by three enhanced product ion scans over mass range of 100–1600 amu. The three most intense peaks were selected for fragmentation which satisfied IDA (Information Dependent Acquisition) criteria. A precursor ion within a 2.5 amu window, once selected for fragmentation, was excluded from detection for 60 s. Curtain gas was set at 15 lbs, nitrogen was used as the collision gas and the ionization tip voltage used was 2000 V.

**Supplementary Figure Legends**

**Figure S1 The ITC analyses showed no binding of chitin polymers to TCLL**

The thermogram of chitobiose (A), chitotetrose (B) and chitohexose (C) to TCLL were not fitted to the experimental data which shows no interaction of these polymers with TCLL.

**Figure S2** **Typical MS/MS spectra from MALDI TOF-TOF analysis and corresponding sequence of representative peptides**

CID MS/MS spectra of trypsin and Glu-C endoproteinase digested TCLL obtained from MALDI TOF/TOF mass spectrometer. A. MS/MS spectrum of m/z 1420.61. B. MS/MS spectrum of m/z1862.92. C. MS/MS spectrum of m/z 911.43. D. MS/MS spectrum of m/z 1389.61.

**Figure S3** **Phylogenetic analysis of TCLL**

The tree was constructed by Neighbor-Joining method using MEGA5 program and the evolutionary distances were computed using the Poisson correction method.

**Figure S4** **Superposition of TCLL with homologous structures of the GH18 family**

The ribbon diagram shows the superposition of C^α^ atoms of TCLL (red) (4B15), hevamine (blue) (2HVM) from latex of *Hevea brasiliensis*, PPL2 (yellow) (2GSJ) from *Parkia platycephala* seeds, concanavalin B (green) (1CNV) from *Canavalia ensiformis*, xylanase inhibitor protein I (magenta) (XIP-I) from *Triticum aestivum*, xylanase and alpha-amylase inhibitor protein (cyan) (XAIP) from *Scadoxus multiflorus* (3MU7) and sccts1 from *Saccharomyces cerevisiae* (orange) (2UY2). The superposition shows that the overall structure is conserved except some loop regions shown by arrows.

**Figure S5 Electrostatic surface potential map of TCLL with other chi-lectins**

TCLL (4B15), HCgp-39 (1HJW), Ym1 (1E9L), YKL-39 (4AY1), SI-CLP (3BXW), SPG-40 (2DSZ), SPC-40 (2DPE), SPS-40 (2DSU) and IDGF-2 (1JND) displaying chitin binding groove. Electrostatic potential was calculated by Pymol and is colour-coded on the surface from blue (∼63) to red (∼63). Only HCgp-39, YKL-39, SI-CLP, SPS-40 and SPG-40 has appropriate groove and chitin fragment binds at this groove. TCLL displays more negative site and has deep pocket like structure. Ym1, SPC-40 and IDGF-2 do not have well defined cavity.

**Figure S6 Sequence alignment of TCLL with other chitinase/chitinase like protein from tamarind**

Alignment of TCLL with tamarinin and chitinase from tamarind. The conserved residues are represented in black background and the key active site residues for chitinase activity are represented by arrows. The alignment was done using the program CLUSTALW and figure was prepared using ESPRIPT.

**Table S1 Hydrogen bond network of GlcNAc with TCLL residues in S1 and S2 pockets**

**S1 S2**

**GlcNAc atoms TCLL Water TCLL TCLL Water TCLL**

O1 W226

O3 Arg152 NH1 Glu132 N

Arg152 NH2 W38

O4 Arg152 NH2 Tyr167 O

W38 Tyr168 O

W93 Glu132 OE2

O5 W20 Gln74 OE1 Tyr167 OH

W20 Tyr121 O, N W226

W301

O6 Glu119 O W301

` W189 Lys171 NZ

O7 W74 Asp123 OD1 W123

W68 Gln74 NE2 W129

W68 Tyr121 O

N2 W148

**Table S2 Alignment of the two consensus regions in GH18 family plant chitinases and two conserved non-proline cis-peptide bonds**

Consensus sequence region two non-proline cis-peptide bonds

β3 β4 β2 β8

TCLL 78 IKILLQIGQVT 123 DGIDIASV 41 EE 256 FD

Hevamine 72 IKVMLSLGGGI 120 DGIDFDIE 31 AF 255 WS

PPL2 72 IKVMLSIGGGA 120 DGVDFDIE 31 AF 255 WD

ConB 77 VKVFLALGGPK 124 DGIHFDIQ 34 SF 265 WN

Xaip-Ii 68 VKVLLSIGGPA 119 DGIDFHIE 33 GF 253 WD

XIP-I 71 VPVSLSIGGYG 121 DGVDLFLE 36 SF 256 WD

Sccts1 101 KKVLLSLGGAS 150 DGFDFDIE 59 SF 285 WD
